# The impact of influenza and pneumococcal vaccination on antibiotic use: an updated systematic review and meta-analysis

**DOI:** 10.1186/s13756-023-01272-6

**Published:** 2023-07-14

**Authors:** Lotte van Heuvel, John Paget, Michel Dückers, Saverio Caini

**Affiliations:** 1 Netherlands Institute for Health Services Research (Nivel), Otterstraat 118, 3513 CR Utrecht, The Netherlands; 2 ARQ Centre of Expertise for the Impact of Disasters and Crises, Diemen, The Netherlands; 3 Faculty of Behavioural and Social Sciences, University of Groningen, Groningen, The Netherlands

**Keywords:** Vaccination, Influenza, Pneumococcal disease, Antibiotic use, Antibiotic prescriptions, Antimicrobial resistance, Systematic literature review

## Abstract

**Background:**

Vaccination can prevent bacterial and viral infections that could otherwise increase the chances of receiving (unnecessary) antibiotic treatment(s). As a result, vaccination may provide an important public health intervention to control antimicrobial resistance (AMR).

**Objectives:**

Perform a systematic literature review to better understand the impact of influenza, pneumococcal and COVID-19 vaccination on antibiotic use, and to identify differences in effect between world regions and study designs.

**Methods:**

We performed a systematic literature review and meta-analysis which updated previous literature reviews with new data from 1 October 2018 to 1 December 2021. The study focuses on randomised controlled trials (RCTs) and observational studies. Results from the meta-analysis of RCTs were stratified by WHO region and age group. Vote counting based on the direction of effect was applied to synthesize the results of the observational studies.

**Results:**

Most studies are performed in the WHO European Region and the Region of the Americas in high-income countries. RCTs show that the effect of influenza vaccination on the number of antibiotic prescriptions or days of antibiotic use (Ratio of Means (RoM) 0.71, 95% CI 0.62–0.83) is stronger compared to the effect of pneumococcal vaccination (RoM 0.92, 95% CI 0.85–1.00). These studies also confirm a reduction in the proportion of people receiving antibiotics after influenza vaccination (Risk Ratio (RR) 0.63, 95% CI 0.51–0.79). The effect of influenza vaccination in the European and American regions ranged from RoM 0.63 and 0.87 to RR 0.70 and 0.66, respectively. The evidence from observational studies supports these findings but presents a less consistent picture. No COVID-19 studies were identified.

**Conclusion:**

We find that both RCTs and observational studies show that influenza vaccination significantly reduces antibiotic use, while the effect of pneumococcal vaccination is less pronounced. We were unable to study the effect of COVID-19 vaccination and no clear regional patterns were found due to the high heterogeneity between studies. Overall, our data supports the use of influenza vaccination as an important public health intervention to reduce antibiotic use and possibly control AMR.

**Supplementary Information:**

The online version contains supplementary material available at 10.1186/s13756-023-01272-6.

## Background

Antimicrobial resistance (AMR) is a major global public health issue and will become a growing problem in the future. It has been estimated that almost 5 million deaths were associated with bacterial AMR in 2019 [1] and that this number will rise to 10 million deaths attributable to drug-resistant bacteria per annum by 2050 [2]. Resistant bacteria are a problem in all regions of the world, though sub-Saharan Africa and south Asia have experienced the highest burden of AMR in 2019 [1]. One way to tackle AMR is to focus on reducing the incidence and spread of infections, as first described in the World Health Organization’s (WHO) Global Action Plan on AMR in 2015 [3].

Vaccination is a measure to reduce the incidence and spread of infections, in the first place by preventing infectious diseases and reducing the prevalence of primary bacterial and viral infections that are often treated with (empiric) antimicrobial therapy [3]. Vaccines can help reduce the use of antimicrobials for both appropriate (e.g. bacterial co-infections due to a viral infection) and inappropriate treatments [4]. Licensed vaccines that are recommended by WHO for their potential impact on AMR include: pneumococcal conjugate vaccines (PCV), typhoid conjugate vaccines (TCV), *Haemophilus influenzae* type b (Hib) vaccines, influenza vaccines, rotavirus vaccines, and measles vaccines [5].

Two recent systematic literature reviews by Buckley et al. (2019) and Doherty et al. (2020) have highlighted the impact of vaccination on antimicrobial use [6, 7]. Both reviews aimed to assess the effect of vaccination on the use of antibiotics, focusing on influenza and pneumococcal vaccination due to the availability of data. The majority of included studies concluded that vaccination with influenza and pneumococcal vaccines significantly reduced antimicrobial consumption after vaccination. The Buckley and Doherty studies found there was a lack of data for all world regions. They presented effect measures for randomised controlled trials (RCTs) but the effect in observational studies was less well studied and remains unclear [6, 7].

WHO has stressed the need to expand and share knowledge and awareness about the impact of vaccines regarding AMR [5], with one of the priority actions for researchers being an assessment of the impact of vaccination on AMR including antimicrobial use. Our study therefore aims to further understand the impact of influenza, pneumococcal and COVID-19 vaccination on antibiotic use. This systematic literature review and meta-analysis provides an update (+ 3 years) to the current evidence (Buckley and Doherty reviews), to identify differences in effect between world regions and study designs.

## Methods

### Search strategy and selection criteria

This literature review and meta-analysis is an update of two previous reviews [6, 7] and we included new studies published from 1 October 2018 to 1 December 2021, covering publications not captured by the data collection period in the former reviews. Papers published before 1 October 2018 that had been included by Buckley, Doherty and colleagues were added to the reference list of relevant papers and further assessed for eligibility. The Buckley study [6] included eligible papers published from January 1998 to March 2018 and the Doherty study [7] from January 2000 to October 2018.

Our study focused on vaccines for influenza and pneumococcal disease for all patient populations, and we also included COVID-19 vaccines in the search strategy. Papers were eligible for inclusion that (1) showed the effect of influenza, pneumococcal, or COVID-19 vaccination (any vaccine type) on the number of antibiotic prescriptions or the days of antibiotic use (individual-based studies), or (2) estimated the effect of national influenza, PCV or COVID-19 vaccines on antibiotic consumption in the community (ecological studies). We only included studies that focused on the direct protection of vaccination in the meta-analysis e.g. we excluded papers that studied the herd effect of vaccination. Randomised, observational, modelling and costs studies were included that reported the effect of vaccination on antibiotic use. See Box 1 for a detailed PICOS statement.

**Box 1:** PICOS statement

*Population*
Children, adults and elderly of all ages and with or without chronic conditions
*Intervention*
Pneumococcal, influenza and/or COVID-19 vaccination (all vaccine types)
*Comparison*
Vaccinated versus unvaccinated (placebo) or vaccinated with other vaccine type
*Outcome*
Antibiotic use (all types) or antibiotic prescriptions
*Study design*
Randomised controlled trials, observational studies (case-cohort, control, cross-sectional), modelling studies, cost studies

We aimed to understand the effect of vaccination on the frequency of antibiotic use and we therefore excluded papers that only focused on the change in one type of antibiotic prescription over time. One reason for this is that revisions of antibiotic prescription guidelines and/or policies can also influence the type of antibiotics prescriptions over time. Studies were also excluded that assessed the effect of vaccination on non-susceptible infections or antimicrobial susceptibility (i.e. in which vaccine use was linked to the percentage of isolates testing non-susceptible to certain antimicrobials).

The search was undertaken on 1 December 2021. We adopted a search strategy similar to the previously published reviews by Buckley [6] and Doherty [7], using a combination of a term including #1 vaccination AND #2 diseases AND #3 antimicrobials (see Box 2). The literature search was conducted in the databases of Ovid Medline, Embase, PubMed and the Cochrane Library. We limited the search to the following languages: Dutch, English, French, German, Italian, Portuguese, Russian, and Spanish. We included both peer-reviewed, published papers and conference abstracts.

**Box 2:** Search strategy

*#1 vaccination*
((Anti-bacteri* or antibacteri* or bacteri*) OR (anti-viral or antiviral or viral or anti-virus* or antivirus* or virus*)) AND (vacc* or immun*)
*#2 diseases*
Pneumo* OR influenza* or flu or flus or H1N1 or H5N1 or H3N2 or Yamagata or Victoria OR coronavirus or corona or COVID-19 or SARS-CoV-2 or COVID
*#3 antimicrobials*
(Antibiotic* or anti-biotic* or abx or antibacterial* or anti-bacterial* or antiinfective* or anti-infective* or antimicrobial* or anti-microbial* or antimycobacterial* or anti-mycobacterial*) AND ((drug or drugs) AND (prescrib* or prescription* or usage? or “use” or uses or utilis* or utiliz*))

Duplicate records were removed using Endnote X9. Title and abstract screening was performed independently by two reviewers (LvH and SC) using Rayyan [8], a tool to organize and manage collaborative systematic reviews, with blind on (i.e. decisions and labels of any collaborator are not visible to others). Studies were selected as ‘included’, ‘excluded’ or ‘maybe’ and any conflicts were discussed until consensus was reached. A third researcher (JP) was invited to participate in these discussions whenever necessary to reach consensus. The full texts of all studies highlighted as ‘included’ were retrieved and read in full copy to assess eligibility for inclusion. Other literature reviews on the topic were not included, but their reference lists were screened for possible eligible papers.

Data from the eligible papers were extracted by one reviewer (LvH) and cross-checked by a second reviewer (SC). The data extraction template was first tested independently by both researchers while analysing data from a random sample of three eligible papers, and any issues were resolved before continuing the data extraction process. An attempt was made to contact authors to obtain data on the antibiotic use data collection methods. Data extracted from RCTs included in the two published literature reviews [6, 7] were cross-checked by one reviewer (focused on estimate of effect), although data from observational studies were only checked if something was unclear.

The following information (based on the data extraction form by Buckley [6]) was extracted from the included papers: first author; journal; publication year; data collection period (and follow-up); country of study; study design; study population including setting, sample size, health conditions and age distribution; data type (individual or ecological); infectious disease; vaccine type; type of antibiotics; outcome measure; estimate of effect (RR, RoM, VE etc.) and corresponding measure of statistical uncertainty (i.e. 95% confidence interval (CI), standard errors or exact p-values); direction of effect (positive, negative or no effect); antibiotic use data collection method (register, medical records, questionnaire, interviews etc.); and funding source.

### Data analysis

This study followed the Preferred Reporting Items for Systematic Reviews and Meta-analyses (PRISMA) reporting guideline, see Additional file 1, [9]. Methodological quality of the included studies was assessed on the basis of the Cochrane risk-of-bias tool for randomised trials (RoB 2) [10] and the Cochrane tool for assessing risk of bias in non-randomised studies of interventions (ROBINS-I) [11]. The risk of bias was assessed by one reviewer (SC) and cross-checked by a second reviewer (LvH). We also extracted evidence of the risk of bias as assessed by Buckley and colleagues [6] using similar tools including RoB 2, ROBINS-I and EPOC.

For each meta-analysis, data from the RCTs were combined and presented using a random-effects model to estimate the summary effect size (Risk Ratio (RR) and Ratio of Means (RoM)) and its 95% CIs. Results were stratified by WHO region: European Region (EUR), Region of the Americas (RAM), and the Western Pacific Region (WPR)^1^. No RCT data were available for the WHO African Region (AFR), South-East Asian Region (SEAR) or the Eastern Mediterranean Region (EMR). If this method was not possible due to data availability, results were stratified by age group (in a similar manner to the Buckley study). Between-study heterogeneity was estimated by using the I^2^ statistics, with large heterogeneity across studies defined as I^2^ > 50% threshold. Because of a limited study size, we were unable to apply subgroup analysis (except the pre-planned one according to WHO region and age group) and perform a meta-regression to explore potential sources of heterogeneity.

We used vote counting based on the direction of effect [12] to perform a meta-analysis of the effect of vaccination on antibiotic use as reported in observational studies. The vote counting method was used instead of formal random effects meta-analysis due to extreme variability in the studies’ endpoints, statistics and ways of reporting (i.e. differences in the magnitude of the association and measures of statistical uncertainty etc.). Vote counting was implemented to compare the number of studies showing a reduction of antibiotic use after vaccination (positive effect) and the number of those showing an increase of antibiotic use (negative effect). This method does not provide information on the magnitude of effects and does not account for differences in the relative sizes of the studies [12].

As part of the vote counting method, the direction of effect was defined by one reviewer (LvH) as ‘positive’, ‘negative’ or ‘no effect’ and any conflicts were discussed with two other reviewers (SC and JP). To overcome the absence of a formal effectiveness threshold, the researchers (LvH, SC and JP) discussed a cut-off value for studies reporting minor effect differences and decided to treat studies reporting a < 2.5% effect difference in the percentage of antibiotic prescriptions between vaccinated and unvaccinated or pre- and post-vaccination were considered as ‘no effect’. Statistical significance was not considered in this categorization as priority was given to the magnitude of the effect. A p-value for the probability of obtaining the observed distribution of studies with positive and negative effects was calculated using the sign test [13]. Studies with conflicting or unclear effect direction could not be included in the sign test. The results are presented in an effect direction plot based on the Cochrane Handbook [12] and updated by Boon and Thomson, 2021 [13]. Study quality is also highlighted in the effect direction plot by row colour (green, yellow or red).

All statistical analyses were performed using Stata software, version 17, and R software, version 4.0.0. *P*-values lower than 0.05 were considered statistically significant.

## Results

**Fig. 1:**
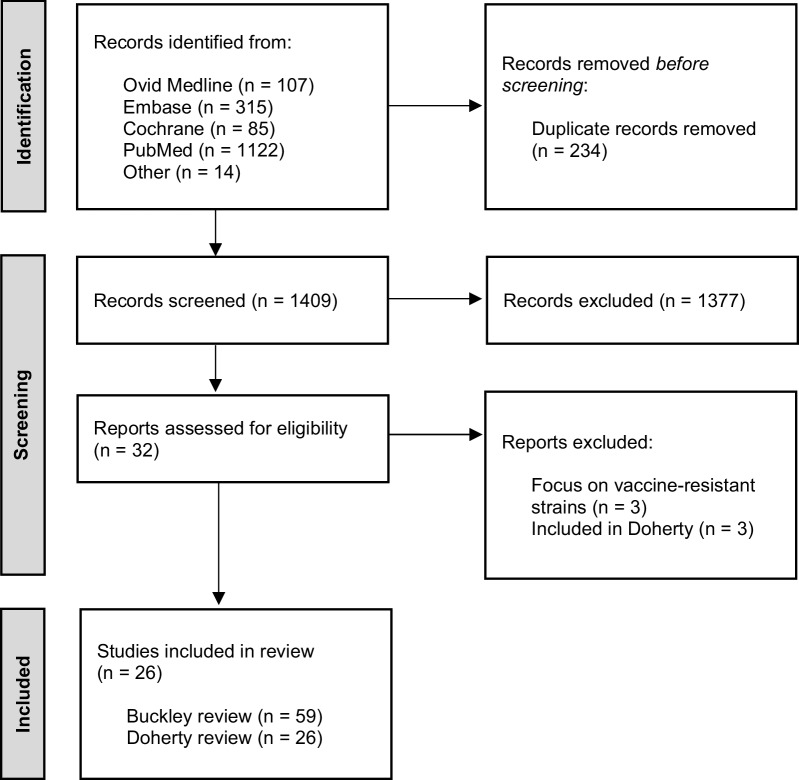
PRISMA study flow diagram

The electronic database searches identified 1409 unique articles after removal of duplicates. The majority of records (n = 1377) were excluded based on title and abstract screening, i.e. not relating to influenza, pneumococcal or COVID-19 vaccination and/or antibiotic use or AMR. A total of 32 full-text reports were assessed and 26 eligible studies were included in the review (see Fig. 1), including the Buckley and Doherty literature reviews. Our analysis includes 87 articles identified by Buckley and colleagues [6] and 26 articles by Doherty, Hausdorff and Kristinsson [7], with overlap between both studies. Overall, our review includes a total of 24 new studies, 59 Buckley and 26 Doherty studies of which 29 RCTs and 69 observational studies. The focus of these studies is on influenza vaccination (n = 16 RCTs; n = 19 observational) or pneumococcal vaccination (n = 12 RCTs; n = 48 observational) and three papers studied the effect of both vaccines (n = 1 RCT; n = 2 observational). No reports were identified studying the impact of COVID-19 vaccination on antibiotic use.

The included studies present data for different outcome measures: (1) the proportion of people receiving antibiotics, (2) the number of antimicrobial courses or prescriptions per person, and (3) the days of antibiotic use. We have combined the outcome measures (2) and (3) for the purpose of the meta-analysis. We acknowledge that a change in the number of total antibiotic courses also represents and measures a change in the days taking antibiotics. It is of note that most studies reviewed antibiotics prescribed for influenza like illness, upper/lower respiratory tract infections, otitis media or any other respiratory infections related antibiotic courses based on lab confirmed and non-lab confirmed cases.

Among all studies included in this review, data on antibiotic use was collected through: (1) subjective self-report data using qualitative methods e.g. questionnaires, interviews, patient diary or (2) objective data from medical records, databases and registers including pharmacy-dispensing data. The effect of vaccination was often followed in randomised studies for one or two seasons, whereas observational studies included data over a longer study period. For PCV, studies also compared two vaccine types (e.g. PCV7 followed by PCV13) over a period of multiple years.

An overview of the included studies’ characteristics can be found in Additional file 2.

### Randomised studies

Out of a total of 29 RCTs, the majority of studies used patient or parental self-reported data (n = 18), collected data on antibiotic prescriptions through medical records and registers (n = 6), performed interviews with parents/caregivers (n = 2), and for 3 studies the methods were unknown.

The results are presented separately for RCTs that studied the effect of influenza or pneumococcal vaccines.

#### Influenza vaccination

A total of 17 RCTs studied the effect of influenza vaccination on antibiotic use and prescriptions. The majority of studies were performed in WHO EUR (n = 7) and in WHO RAM (n = 5), e.g. in the United States and Canada. The study population mainly consisted of children aged < 5 years (n = 7) or older children (n = 4), with only two studies performed in the general adult population (age 18–64 years) and two studies among older adults > 65 years. We excluded two RCTs from the meta-analysis (included in the Buckley analysis) that looked at the number of antibiotic prescriptions among household contacts of vaccinated children i.e. herd effect of vaccination [14, 15].

**Fig. 2:**
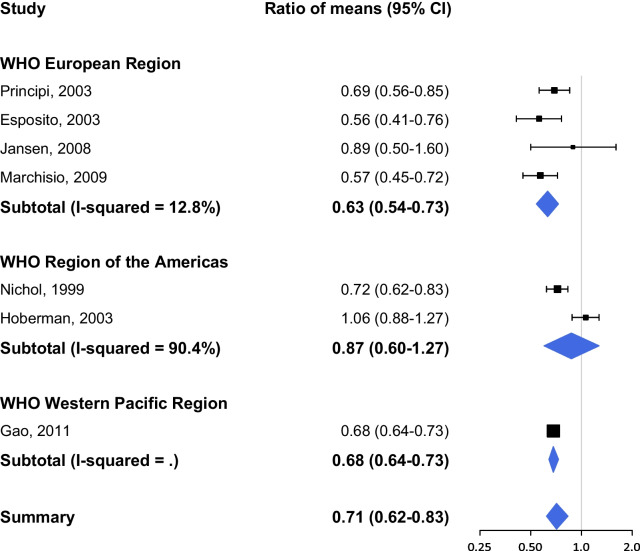
Proportion of people receiving antibiotics after influenza vaccination

**Fig. 3:**
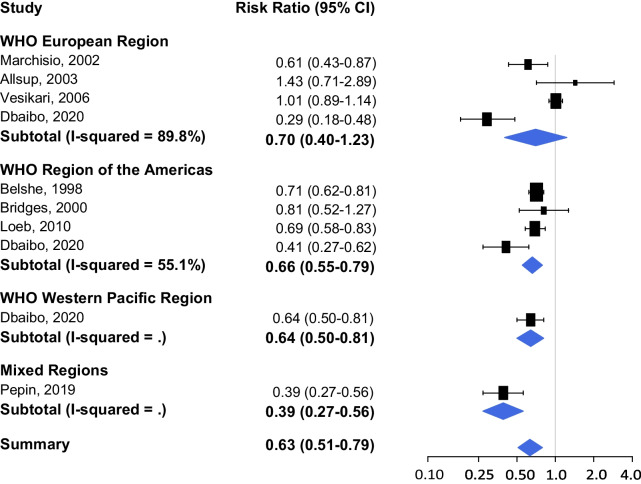
Number of antimicrobial prescriptions or days of antibiotic use after influenza vaccination

Figures 2 and 3 show the results of the pooled analysis for two different outcome measures respectively: (1) the proportion of people receiving antibiotics, and (2) the number of antimicrobial prescriptions or days of antibiotic use; stratified by WHO region. The study by Dbaibo et al. (2020) presents data on antibiotic use per region: Europe, Asia Pacific and Central America [16]. The studies in WHO EUR versus WHO RAM are weighted equally for RCTs evaluating (1) the proportion of people receiving antibiotics after influenza vaccination, and the effect is similar (RR 0.70, 95% CI 0.40–1.23 vs. RR 0.66, 95% CI 0.55–0.79; Fig. 2). The overall reduction in (2) antimicrobial prescriptions or days of antibiotic use (RoM 0.71, 95% CI 0.62–0.83; Fig. 3) is less distinct compared to the reduction in the proportion of people receiving antibiotics (RR 0.63, 95% CI 0.51–0.79; Fig. 2).

There was one study in Fig. 3 with a low risk of bias that examined the outcome ‘days taking antibiotics’ [17]. This study by Nichol et al. (1999) showed a 28.1% reduction (95% CI 16.6–38.0%) in the number of days taking antibiotics for febrile illness among healthy, working adults aged 18–64 years in the general adult population who received the live-attenuated influenza vaccine (LAIV) compared to placebo [17].

Three RCTs presented no effect (i.e. no statistical significant difference) of influenza vaccination on antibiotic use [18–20]. The study by Allsup and colleagues [18] showed similar levels of antibiotics prescribed for respiratory infections for influenza vaccinated elderly > 65 years compared to placebo (RR 1.43, 95% CI 0.71–2.89). Both studies by Hoberman [19] and Vesikari [20] were performed in children < 36 months and showed no reductions in vaccinated children in antibiotics prescribed (RoM 1.06, 95% CI 0.88–1.27; RR 1.01, 95% CI 0.89–1.14; respectively). The studies by Bridges [21] and Jansen [22] also presented non-significant effects (RR 0.81, 95% CI 0.52–1.27; RoM 0.89, 0.50–1.60; respectively).

Overall, there is a high level of heterogeneity between studies (I^2^ > 50) in the WHO European and American Regions and the WHO region-stratified meta-analysis shows a high variability in the magnitude of effect.

#### Pneumococcal vaccination

We included 13 RCTs which studied the effect of pneumococcal vaccination on antibiotic use. Only two studies present data regarding the outcome ‘proportion of people receiving antibiotics’ after pneumococcal vaccination: one RCT in elderly patients with COPD [23] and one RCT in children < 12 weeks old [24]. We were not able to perform a meta-analysis for this outcome measure due to limited study availability. We identified 11 RCTS studying ‘the number of antimicrobial prescriptions or days of antibiotic use’ after pneumococcal vaccination. Stratifying the results per WHO region was not possible for this outcome because of the similarities in country of study, e.g. 8 RCTs in WHO EUR, 1 in WHO RAM and 1 in WHO WPR, and we therefore stratified the analysis by age group similar to the Buckley review. The country of study for one paper was unclear. The study population included children (n = 8), patients with COPD (n = 2) and older adults > 65 years (n = 1). The studies covered different vaccine types including PCV7, PCV9, PCV13, PPV23, and PHiD-CV10 (pneumococcal *Haemophilus influenzae* protein D conjugate vaccine).

**Fig. 4:**
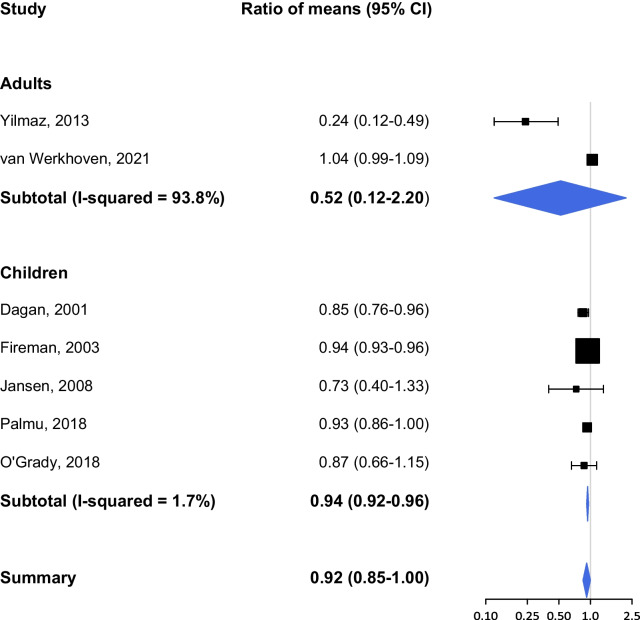
Number of antimicrobial prescriptions or days of antibiotic use after pneumococcal vaccination

Results from the pooled analysis showed a minor, yet nearly statistically significant effect of pneumococcal vaccination on the number or days of antimicrobial prescriptions (RoM 0.92, 95% CI 0.85–1.00; Fig. 4). The effectiveness of pneumococcal vaccination is higher among adults versus children (RoM 0.52, 95% CI 0.12–2.20 vs. RoM 0.94, 95% CI 0.92–0.96). There is large heterogeneity between the two studies [25, 26] performed among adults (I^2^ = 93.8%). The study by Yilmaz et al. (2013) showed a large and significant effect of PPV23 among older adults with COPD (RoM 0.24, 95% CI 0.12–0.49), but these results do not strongly influence the overall effect (weight 1.17%). Van Werkhoven et al. (2021) studied the effect of PCV13 on the total number of antibiotic prescriptions among healthy adults ≥ 65 years and found no significant differences compared to placebo (RoM 1.04, 95% CI 0.99–1.09).The outcome by van Werkhoven [26] is of high quality and more in line with the pooled estimate for children.

One study included in the meta-analysis also looked at the effect of PCV9 on the outcome ‘days of antibiotic use’ in children aged 12–35 months [27]. Dagan and colleagues (2001) observed reductions of 15–17% in upper/lower respiratory tract infections and otitis media resulting in an overall reduction of 17% in antibiotic days [27]. Furthermore, three other studies present a non-significant effect: Janssen [22] RoM 0.73 (95% CI 0.40–1.33), Palmu [28] RoM 0.93 (95% CI 0.86–1.00), and O’Grady [29] RoM 0.87 (95% CI 0.66–1.15).

### Observational studies

**Fig. 5:**
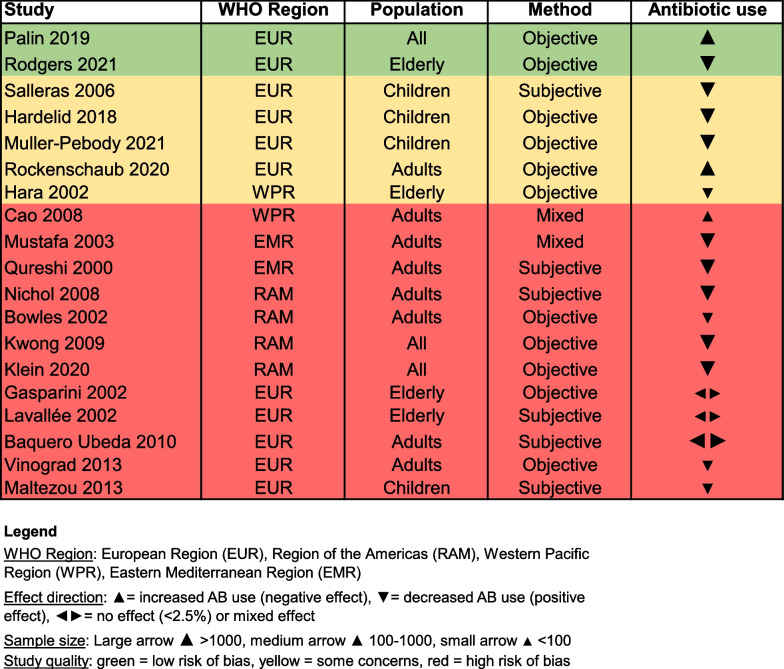
The effect of influenza vaccination on antibiotic use in observational studies

**Fig. 6:**
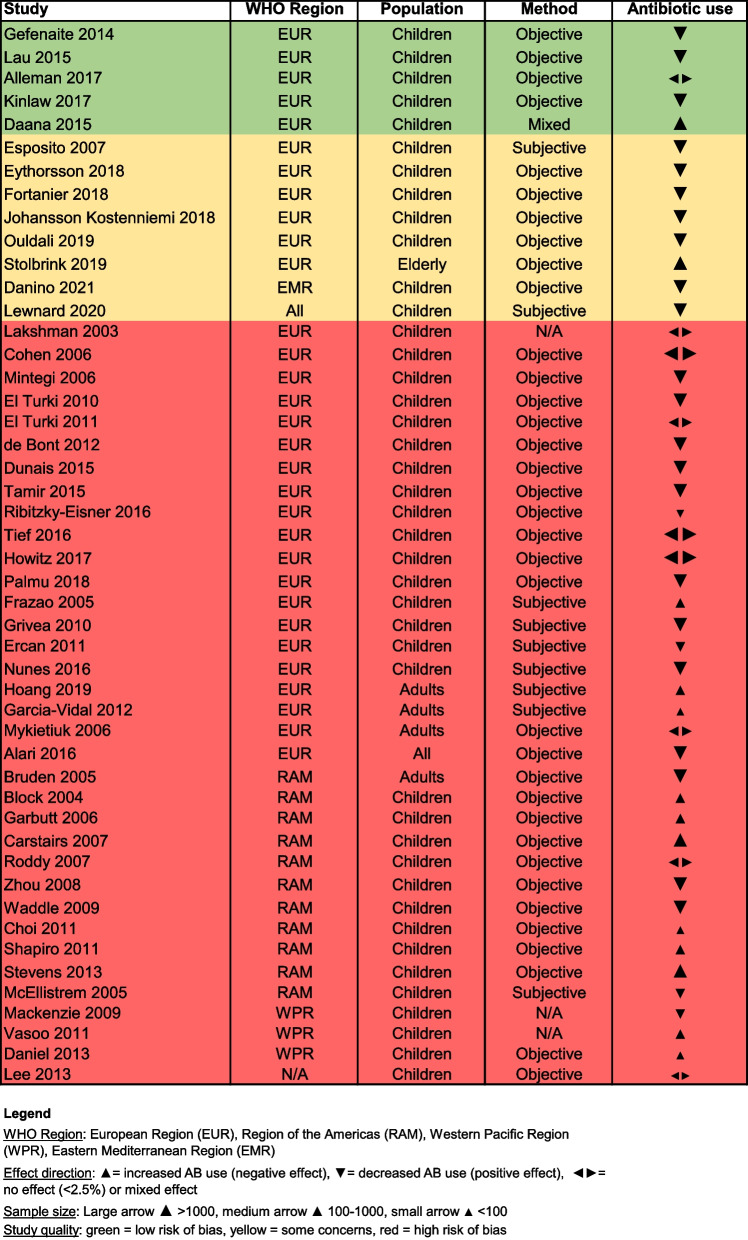
The effect of pneumococcal vaccination on antibiotic use in observational studies

Out of the 69 included observational studies, there were a total of 19 influenza papers and 48 papers that studied the effect of pneumococcal vaccination on antibiotic use. Two studies combined the influenza and pneumococcal vaccine (PCV and PPV) to show the result in vaccinated versus unvaccinated people [30, 31]. A total of 42 studies made use of a large database and included a large sample size (n > 1000), while there are only 6 studies with a small sample size (n < 100). The majority of the studies were performed in WHO EUR (n = 42), followed by WHO RAM (n = 15) and other regions including WHO WPR and the WHO Eastern Mediterranean Region (EMR), see Figs. 5 and 6. Most of the observational studies for influenza were performed in adults and the elderly in comparison to the randomised studies which were mainly performed in children. For PCV, almost all observational studies were performed in children (88%). A detailed overview of the studies is shown in Additional file 2.

A total of 16 out of the 69 observational studies used a subjective method to collect antibiotic use data, while a majority of the studies collected data through objective methods (n = 46). The antibiotic use data collection method was unclear for 4 observational studies and 3 studies used mixed methods. The data collection methods per study are presented in Figs. 5 and 6.

Most observational studies had a high risk of bias (red colour), with only 7 studies assessed as at low risk of bias (green colour) and 13 studies at moderate risk (yellow colour), as shown in Figs. 5 and 6. It is of note that almost all high and moderate quality studies were performed in WHO EUR. There are two influenza studies with a low risk of bias that showed contrasting results, namely that vaccinating patients > 65 years against influenza reduces antibiotic prescriptions by 14% (Hazard Ratio 0.86, 95% CI 0.81–0.9) to treat respiratory infections [32] compared to a 3–17% increased likelihood for the general population to receive an antibiotic prescription for sinusitis and lower respiratory tract infections respectively [33]. Among the five pneumococcal studies with a low risk of bias, three studies showed a positive effect of PCV7 and PCV13 vaccination among children by reducing antibiotic prescriptions up to 19% per month [34–36], while one PCV7 study showed a 17% increase in the proportion of children receiving antibiotics when the vaccination rate increased [37], and one study showed only a minor change (< 2.5%) in antibiotic use among children after the introduction of PCV7 (antibiotic use increased from 14.8 to 16.4%) and subsequently PCV13 vaccination (antibiotic use decreased from 16.4 to 14.4%) [38].

For the influenza studies, 13 papers reported a positive effect, 3 reported a negative effect and 3 papers reported no effect. According to the vote counting method, the null hypothesis of no effect of the influenza vaccine on antibiotic use can be rejected with a p-value equal to 0.021. In comparison, 26 papers reported a positive effect of pneumococcal vaccination while 13 reported a negative effect and 9 studies showed no effect. Likewise, the null hypothesis of no effect of pneumococcal vaccination on antibiotic use can be rejected with a p-value of 0.037. Thus, we can reject both null hypotheses (at 0.05 significance level) and state that the evidence from the observational studies is in favour of the influenza and pneumococcal vaccine being effective in reducing antibiotics use and prescriptions, although we cannot quantify this effect.

## Discussion

This updated literature review confirms earlier findings which showed that influenza and pneumococcal vaccines can reduce antibiotic use (all types), including the proportion of people receiving antibiotics, the number of antimicrobial prescriptions and the days of antibiotic use. No clear regional patterns were found due to the high heterogeneity between studies. Importantly, our meta-analysis shows a more nuanced effect of pneumococcal vaccination on antibiotic use compared to influenza vaccination, where a significant reduction was observed. This study provides a more comprehensive overview and updated meta-analysis regarding pneumococcal vaccination and this helps us to have a better understanding of the differences in effect between a viral and bacterial vaccine on antibiotic use.

Our study supports the meta-analysis by Buckley [6] and presents a positive effect of influenza and pneumococcal vaccination on antibiotic use. The overall effect is similar between both studies for the outcome ‘number of antimicrobial prescriptions or days of antibiotic use’ after (1) influenza and (2) pneumococcal vaccination: (1) RoM 0.71 (95% CI 0.62–0.83) versus RoM 0.75 (95% CI 0.62–0.90) in Buckley, and (2) RoM 0.92 (95% CI 0.85–1.00) versus RoM 0.93 (95% CI 0.87–0.99) in Buckley. We have combined both outcome measures in comparison to Buckley as we consider that a reduction in the number of days of antibiotic use presents a reduced total number of antimicrobial prescriptions. The effect of influenza vaccination for the outcome measure ‘proportion of people receiving antibiotics’ is stronger compared to Buckley: RR 0.63 (95% CI 0.51–0.79) versus RR 0.79 (95% CI 0.65–0.97) in Buckley. Overall, the updated meta-analysis shows a somewhat stronger effect of influenza and pneumococcal vaccination on antibiotic use compared to the Buckley review.

The Doherty study [7] also confirms that the evidence on influenza and pneumococcal vaccination strongly indicates a reduction of antibiotic use after vaccination, whereas 23 out of 26 included studies showed significant reductions in antimicrobial use. They note that overall prescribing rates (associated to otitis media and upper respiratory tract infections) were significantly reduced both in vaccinated individuals and at a population level. Doherty and colleagues have not performed a meta-analysis but their main conclusion is in line with the Buckley study and our updated review.

It is of note that the impact of both vaccines on antibiotic use is different, with the effect of influenza vaccination being remarkably stronger (21%) compared to pneumococcal vaccination (RoM 0.71, 95% CI 0.62–0.83 vs. RoM 0.92, 95% CI 0.85–1.00). The strong effect of influenza vaccination on reducing antibiotic use is surprising as an influenza infection should not be treated with antibiotics except for secondary (bacterial) infections due to the influenza disease. One possible explanation for the effect difference is the inherent characteristics of the two vaccines, including vaccinology between viral and bacterial vaccines and pathogenesis of the diseases. While PCV directly impacts the prevalence of drug-resistant pneumococcal disease, influenza vaccines work via a secondary effect through a reduction in acute respiratory infections and influenza-like illness that often lead to the (inappropriate) use of antibiotics [4]. In addition, an influenza infection can increase the risk of secondary bacterial infections such as pneumonia and otitis media, which then require antibiotic treatment [39]. The higher impact of influenza vaccines on antibiotic use might be related to the annual recurrence of influenza epidemics due to the high transmissibility of the disease compared to pneumococcal disease.

Despite these factors, the differences in effect between the two vaccines cannot be explained by the efficacy of the vaccines in preventing infections. The vaccine effectiveness (VE) of influenza vaccines (inactivated influenza vaccines (IIV) and LAIV) against laboratory-confirmed influenza virus infection ranges from 30 to 60% while the VE for pneumococcal vaccination (PCV7 and PCV9) among infants is considerably higher (approximately 70–95%) in reducing the risk of invasive pneumococcal disease [40]. A poor match between seasonal influenza vaccines and the circulating strains can result in a lower VE. Other factors that may influence VE are age, sex, health status, prior history of vaccination, type of vaccination, and study setting [40]. We have therefore included papers covering different study populations, age groups, settings and vaccine types. For influenza these are IIV including Trivalent or Quadrivalent Influenza Vaccines or LAIV, and for pneumococcal vaccines the studies included PCV7, PCV9, PCV13, PPV23, and PHiD-CV10 vaccines. Regardless of the lower effect of PCV on antibiotic use compared to influenza vaccination, this effect should not be overlooked as a 6% decrease in the use of antibiotics can be substantial because of the high incidence of pneumococcal disease and antibiotic use worldwide.

In this review we indicate that observational studies present a more nuanced and less consistent effect of vaccination on antibiotic use compared to randomised studies. There has been debate about the hierarchy of evidence resulting from different study designs, and it is conventionally assumed that RCTs are the ‘gold standard’ in clinical research [41]. Looking at the strength of evidence based on randomised versus observational studies, one might argue that vaccination is more effective in experimental circumstances compared to real-world circumstances [42]. However, ignoring the results of over 60 observational studies, despite the quality of many of the studies, would not reflect the whole picture and we must therefore interpret the effect of vaccination on antibiotic use with caution. It should be noted that the observational studies with the strongest designs and objective measures also point to a decrease of antibiotic use for both influenza and pneumococcal vaccination (see Figs. 5 and 6).

### Strengths and limitations

This study is the most comprehensive and up-to-date systematic literature review since 2018 that assessed the association between influenza and pneumococcal vaccination and antibiotic use. An important strength of our study is that we have included a detailed and more systematic analysis of all observational studies. When interpreting the results of the meta-analysis, it is important to be aware of the heterogeneity between studies in study design, vaccine types, study populations and outcome measures. Furthermore, we need to acknowledge the risk of publication bias and the impact this might have on the overall analysis and specifically the vote counting methods based on the direction of effect (positive, negative or no effect). Studies that present negative results (i.e. no effect or an opposite effect) may be less frequently published [43] and therefore included in our analysis.

Three limitations related specifically to the evidence of the studies included in this review are: (1) the quality of evidence of most of the studies was low or moderate, (2) the type of antibiotics were often not reported, and (3) studies were predominantly performed in high-income countries in the WHO EUR and RAM regions. A total of only 7 out of 69 observational studies and 3 out of 28 RCTs were assessed as having a low risk of bias i.e. presented high quality evidence. These high quality studies were mainly performed in high-income countries in the WHO EUR region. The lack of high quality studies can be related to the fact that antibiotic use is often measured as a secondary outcome measure. In addition, not all studies reported the type of antibiotics that were studied and hence we included all antibiotic types. It therefore remains unclear whether influenza and pneumococcal vaccines specifically impact antibiotic usage for respiratory infections. In the future it will be important for studies to include more detail on the type of antibiotics used.

The quality of the included studies might also be influenced by the quality of the data collection methods used to assess antibiotic use. We identified two broad approaches to measure antibiotic use, either through collecting objective or subjective data. It is of note that the majority of observational studies used objective methods (77%) compared to subjective data collection methods used mainly in RCTs (77%). It is likely that these data collection methods influence the estimates, however research shows that well-designed observational studies (cohort or case–control studies) did not systematically over-estimate the effect estimates in comparison to RCTs [44]. This refers back to the discussion about the hierarchy of evidence between RCTs and observational studies [41], and our study highlights the importance of also assessing observational studies. We can contemplate whether RCTs that use subjective antibiotic use data (with the possibility of recall bias) are of higher quality than observational studies that use objective data. Nevertheless, we chose to report all available studies regardless of study quality to refrain from selective outcome reporting.

Our review found that vaccine studies on this topic were mainly performed in WHO EUR and RAM regions and the studies were therefore predominantly performed in high-income countries that have extensive experience with effective vaccination campaigns, as well as antibiotic stewardship policies. This limitation might be inherent to the search strategy which was limited to languages readable by the researchers, and this could have resulted in selection bias as we did not include studies in Asian languages. Research shows that the highest burden of respiratory infections and AMR is in sub-Saharan Africa and South Asia [1, 45], while this review demonstrates that not much research has been done in low-income countries in these regions. It is not clear whether the effect of vaccination on antibiotic use is similar in countries with other healthcare systems compared to Europe and North-America. Global differences in healthcare systems (and national immunisation programmes) and the (over the counter) availability of antibiotics in many of these countries can strongly influence antibiotic use [46]. We therefore need more research on how healthcare systems and vaccination policies can influence antibiotic use worldwide as well as data from more diverse regions including lower-income countries to make the study results more generalizable.

Overall, the findings in this study are positive regarding the impact of influenza and possibly pneumococcal vaccination on antibiotic use but more research is still needed, more specifically on studies where antibiotic use is the primary objective, studies that are performed in a more diverse sample of countries, and studies that include a higher quality of study designs. There is an opportunity for new vaccine trials to focus more on these aspects (e.g. the PCV21 study). It is also important to update this review if and when new studies become available, especially for COVID-19 vaccines and with other new and emerging infectious diseases. Importantly, we identified five new observational studies after the data collection period in favour of influenza and pneumococcal vaccination. The studies showed that influenza vaccination may reduce AMR proportions [47] and antibiotic prescribing among children [48, 49] and low-risk adults [50], and pneumococcal vaccination (PHiD-CV/PCV13) can decrease the frequency of antibiotic use [51].

### Public health implications

Our findings have important implications for public health policies and other international policies in the field of AMR. While vaccination is acknowledged by the WHO and other global health organizations as an important public health intervention, the implementation of vaccines against AMR in national policies remains unclear [52]. Research shows that pneumococcal and influenza vaccination are the most frequently highlighted vaccines in AMR National Action Plans worldwide [52] and this review supports the use of influenza vaccination and possibly pneumococcal vaccination as a public health intervention aimed at addressing antibiotic use and AMR. These interventions need to be integrated into a multi-pronged strategy that takes into account all of the other factors that can reduce antibiotic use over time, such as antibiotic stewardship policies, raised awareness about rational antibiotic use through antibiotic campaigns and/or access to antimicrobials [6, 7].

To further inform AMR policies, it is important to understand the effect of vaccination on AMR. The magnitude of the effect of reduced antibiotic use on AMR is often not studied, and this review therefore focused on the effect of vaccination on antibiotic use specifically. Two studies have shown that a reduction in antibiotic consumption rates significantly reduces macrolide resistance and that the introduction of PCV vaccination slowed the development of AMR. Increasing PCV immunization coverage could even lead to major annual AMR cost savings due to averted antimicrobial purchases and pharmacy costs [53, 54]. These are reasons to favour the increased uptake of pneumococcal vaccination.

It is clear that influenza and possibly pneumococcal vaccination can impact antibiotic use, while there is a lack of studies that analysed the impact of other vaccines. Our literature review found no studies that have looked at the effect of COVID-19 vaccines on antibiotic use, even though the COVID-19 pandemic has seriously impacted AMR over the last years. At the beginning of the pandemic, many hospitalized COVID-19 patients (almost 80%) received antibiotics (even without a bacterial infection) and nearly 30.000 people died that year from antimicrobial resistant infections in the United States [55]. In other countries as well, the prevalence of AMR was high in COVID-19 patients [56] and AMR rates have risen in Iran compared to before the pandemic [57]. It is likely that preventing these infections through vaccination could have significantly impacted antibiotic use and more research is needed to identify the global health impact of COVID-19 vaccines on AMR.

## Conclusion

Overall, our study finds that influenza vaccination can be used as a public health intervention to reduce antibiotic use next to existing antibiotic stewardship policies and other regional, national and international strategies to tackle AMR. Whilst both RCTs and observational studies have shown that influenza vaccination can significantly reduce antibiotic use, the effect of pneumococcal vaccination is less pronounced and the effect of COVID-19 vaccination was not studied. There is large heterogeneity between studies and no clear differences between world regions were found. There is a need for more research to understand the differential impact of influenza and pneumococcal vaccines and to assess the effect of vaccines against new and emerging infectious diseases. Nevertheless, influenza (and possibly pneumococcal) vaccines are important public health interventions to reduce antibiotic use and as an added benefit may also help in the global fight against AMR.

## Supplementary Information

**Additional file 1.** PRISMA Checklist.

**Additional file 2.** Overview of the study characteristics of all studies included in this review.

## Data Availability

All data generated or analysed during this study are included in this published article [and its Additional files].

## Abbreviations

AMR: Antimicrobial resistance
EUR: European Region
EMR: Eastern Mediterranean Region
Hib: *Haemophilus **influenzae* type b
LAIV: Live-attenuated influenza vaccine
PCV: Pneumococcal conjugate vaccines
PHiD-CV10: Pneumococcal *Haemophilus* influenzae protein D conjugate vaccine
PPV: Pneumococcal polysaccharide vaccine
RAM: Region of the Americas
RCTs: Randomised controlled trials
RoM: Ratio of means
RR: Risk ratio
TCV: Typhoid conjugate vaccines
VE: Vaccine effectiveness
WHO: World Health Organization
WPR: Western Pacific Region

## Footnotes

1 To avoid a misconception with antimicrobial resistance (AMR), the region of the Americas was abbreviated as RAM instead of AMR (as used by WHO).
