# Supplementary material for: The impact of influenza and pneumococcal vaccination on antibiotic use: an updated systematic review and meta-analysis
Source: Antimicrob Resist Infect Control. 2023 Jul 14;12:70. doi: 10.1186/s13756-023-01272-6 (PMC10347879; doi:10.1186/s13756-023-01272-6)
Supplement: Supplementary file 2 — Additional file 2. Overview of the study characteristics of all studies included in this review. [file 13756_2023_1272_MOESM2_ESM.docx]

**Randomised studies:**

| Study ID | Country, WHO region, data period | Population, N, data source | Vaccine type,  Antibiotic type | Outcome,  Estimate of Effect (95% CI) | Study quality |
| --- | --- | --- | --- | --- | --- |
| **Influenza vaccine** | | | | | |
| Allsup 2003  (1) | United Kingdom  WHO EUR  1999 – 2000 | Healthy adults aged 65 – 74 years  N = 729  Antibiotic data collection: medical records | Trivalent Influenza Vaccine vs. Placebo  AB types N/A | Proportions of people receiving antibiotics  Risk Ratio 1.43 (0.71 – 2.89) | Very low |
| Belshe 1998  (2) | USA  WHO RAM  1996 – 1998 | Health children aged 15 – 71 months  N = 1602  Antibiotic data collection: parental self-report | Live-Attenuated Trivalent Influenza Vaccine vs. Placebo  AB types N/A | Proportions of children receiving antibiotics  Risk ratio 0.71 (0.62 – 0.81) | Very low |
| Bridges 2000  (3) | USA  WHO RAM  1997 – 1999 | Healthy adults aged 18 – 64 years  N = 2375  Antibiotic data collection: patient self-report | Trivalent Inactivated Influenza Vaccine vs. Placebo  AB types N/A | Proportions of people receiving antibiotics  Risk ratio 0.81 (0.52 – 1.27) | Low |
| Dbaibo 2020  (4) | 13 countries in Europe, Mediterranean, Asia-Pacific, Central, America  All WHO Regions  2011 – 2014 | Healthy children aged 6 – 35 months  N = 12 018  Antibiotic data collection: parental self-report | IIV4 vs. non-influenza control vaccines  AB types N/A | Proportions of children receiving antibiotics  Europe: RR 0.29 (0.18 - 0.49)  Asia Pacific: RR 0.64 (0.50 - 0.81)  Central America: RR 0.41 (0.27 - 0.62) | Moderate |
| Esposito 2003  (5) | Italy  WHO EUR  2000 – 2001 | Children with history of RTIs aged 6 months – 9 years  N = 127  Antibiotic data collection: parental self-report | Trivalent Inactivated Influenza Vaccine vs. Placebo  AB types N/A | Number of antibiotic courses or prescriptions  Ratio of means 0.56 (0.41 – 0.75) | Moderate |
| Gao 2011  (6) | China  WHO WPR  2008 – 2009 | Patients with chronic bronchitis aged ≥60 years  N = 138  Antibiotic data collection: patient self-report | Influenza vaccine vs. unvaccinated | Number of antibiotic courses or prescriptions  Ratio of means 0.68 (0.64 – 0.73) | Low |
| Hoberman 2003  (7) | USA  WHO RAM  1999 – 2001 | Children aged 6 – 24 months  N = 793  Antibiotic data collection: parental self-report | Trivalent Inactivated Influenza Vaccine vs. Placebo  AB types N/A | Number of antibiotic courses or prescriptions  Ratio of means 1.06 (0.88 – 1.27) | Low |
| Hurwitz 2000  (8) | USA  WHO RAM  1996 – 1997 | Household contacts aged 5 – 17 years  N = 59  Antibiotic data collection: parental self-report | Influenza vs. Hepatitis A vaccine  AB types N/A | Number of antibiotic prescriptions per household  VE 88% (P = 0.02) | Acceptable |
| Loeb 2010  (9) | Canada  WHO RAM  2008 – 2009 | Children and adolescents aged 36 months – 15 years  N = 10 985  Antibiotic data collection: parental self-report | Inactivated Seasonal Influenza Vaccine vs. Hepatitis A vaccine  AB types N/A | Proportions of people receiving antibiotics  Risk ratio 0.69 (0.58 – 0.83) | Moderate |
| Marchisio 2002  (10) | Italy  WHO EUR  1999 – 2000 | Children with history of AOM aged 1 – 5 years  N = 133  Antibiotic data collection: parental self-report | Inactivated Virosomal Subunit Influenza Vaccine vs. Unvaccinated  AB types N/A | Proportions of children receiving antibiotics  Risk ratio 0.61 (00.43 – 0.87) | Very low |
| Marchisio 2009  (11) | Italy  WHO EUR  2006 – 2007 | Children with history of AOM aged 1 – 5 years  N = 180  Antibiotic data collection: parental self-report | Inactivated Virosomal-Adjuvanted Subunit Influenza Vaccine vs. Unvaccinated  AB types N/A | Number of antibiotic courses or prescriptions  Ratio of means 0.57 (0.45 – 0.71)  VE 13.2% (P = <0.001) | Moderate |
| Nichol 1999  (12) | USA  WHO RAM  1997 – 1998 | Healthy adults aged 18 – 64 years    N = 4561  Antibiotic data collection: patient self-report | Live-Attenuated Influenza Vaccine vs. Placebo  AB types N/A | Number of days taking antibiotics for febrile illness  Rate reduction 28.1% (16.6% –  38.0%) | High |
| Pepin 2019  (13) | 9 countries in Latin America, Asia, Africa, Europe    WHO RAM, WPR, AFR, EUR  2014 – 2016 | Healthy children aged 6 – 35 months  N = 5436  Antibiotic data collection: N/A | IIV4 vs. Control  AB types N/A | Proportions of children receiving antibiotics  Relative risk 39.20% (26.89 – 56.24) | Moderate |
| Pisu 2005  (14) | USA  WHO RAM  1996 – 1999 | Families of children aged 2 – 5 years  N = 260  Antibiotic data collection: parental self-report | Inactivated Influenza Vaccine vs. Hepatitis A vaccine  AB types N/A | Number of antibiotic courses prescriptions per household  Ratio of means 0.82 (0.34 – 1.98 | Low |
| Principi 2003  (15) | Italy  WHO EUR  2001 – 2002 | Healthy children aged 6 months – 5 years  N = 303  Antibiotic data collection: parental self-report | Inflexal V Intramuscular Virosomal Influenza Vaccine vs. Unvaccinated  AB types N/A | Number of antibiotic courses or prescriptions  Ratio of means 0.69 (0.56 – 0.84) | Moderate |
| Vesikari 2006  (16) | Belgium, Finland, Israel, Spain, UK  WHO EUR  2000 – 2002 | Healthy children aged 6 to <36 months  N = 1784  Antibiotic data collection: parental self-report | Trivalent Influenza Vaccine vs. Placebo  AB types N/A | Proportions of children receiving antibiotics  Risk ratio 1.01 (0.89 – 1.14) | Very low |
| **Pneumococcal vaccine** | | | | | |
| Dagan 2001  (17) | Israel  WHO EUR  Data period N/A | Healthy children aged 12 – 35 months  N = 261  Antibiotic data collection: parental self-report | PCV9 vs. Meningococcus C Conjugate Vaccine  AB for Upper Respiratory Infections, Lower Respiratory Problems, Otitis Media and other | Number of illness episodes resulting in antibiotic use  Rate ratio 0.85 (0.76 – 0.96)  Antibiotic days for URI: RR 0.90 (0.84 – 0.97), for LRI: RR 0.53 (0.45 – 0.62), for OM: RR 0.80 (0.74 – 0.86) | Moderate |
| Fireman 2003  (18) | USA  WHO RAM  1995 – 1999 | Infants aged <2 months  N = 37 868  Antibiotic data collection: Health Insurance Database | PCV7 vs. Meningococcus C Conjugate Vaccine  Oral antibiotic medications (for any  diagnosis) | Number of antibiotic prescriptions  Vaccine reduced prescriptions by 5.7% (4.2% – 7.2%) | Very low |
| Karppinen 2019  (19) | Finland  WHO EUR  2009 – 2010, follow up period until 2012 | Infants and children aged <2 years  N = 424  Antibiotic data collection: N/A | PHiD-CV10 vs. control (Hepatitis A or B vaccine)  AB types N/A | Number of antibiotic prescriptions (for RTIs)  VE 24% (3% – 40%) | Moderate |
| Nieminen 2019  (20) | Finland  WHO EUR  2009 – 2010, follow up period until 2012 | Infants and children aged <19 months  N = 30 527  Antibiotic data collection: Register | PHiD-CV10 vs. control (Hepatitis A or B vaccine)  AB types N/A | Number of antimicrobial purchases (recommended for AOM)  Preterm infants: VE 12% (1% – 21%) | Moderate |
| O’Grady 2018  (21) | Australia  WHO WPR  2012 – 2015 | Children (with bronchitis/lung disease) aged 18 months to <18 years  N = 61  Antibiotic data collection: parental self-report | PHiD-CV10 vs. MenACYW135  AB types N/A | Number of antibiotic courses (prescribed for cough <14 or <28 days)  <14 days: IDR 0.81 (0.61 – 1.09)  <28 days: IDR 0.87 (0.66 – 1.15) | Acceptable |
| Palmu 2014  (22) | Finland  WHO EUR  2009 – 2011 | Children aged 6 weeks – 18 months  N = 47 366  Antibiotic data collection: Register | PHiD-CV10 vs. Hepatitis A or B Vaccine  Amoxicillin without and with enzyme inhibitor clavulanic acid, phenoxymethylpenicillin, cefuroxime, cefaclor,  sulfadiazine and trimethoprim, clarithromycin, azithromycin | Number of antibiotic prescriptions  Rate ratio 0.93 (0.87 – 0.99) | Moderate |
| Palmu 2018  (23) | Finland  WHO EUR  2009 – 2012 | Infants and children aged <19 months  N = 30 527  Antibiotic data collection: Register | PHiD-CV10  Amoxicillin without and with enzyme inhibitor clavulanic acid, phenoxymethylpenicillin, cefuroxime, cefaclor,  sulfadiazine and trimethoprim, clarithromycin, azithromycin | Number of antimicrobial purchases (recommended for AOM)  VE 7% (0% – 14%) | High |
| Steentoft 2006  (24) | Denmark  WHO EUR  Data period N/A | Adults with COPD  N = 49  Antibiotic data collection: patient self-report | Pneumovax 23-Polyvalent Pneumococcal Vaccine vs. Unvaccinated  AB types N/A | Proportions of people receiving antibiotics  Risk ratio 1.05 (0.72 – 1.51) | Very low |
| Van Gils 2009  (25) | Netherlands  WHO EUR  2005 – 2008 | Infants aged <12 weeks  N = 1003  Antibiotic data collection: parental self-report | CRM197-PCV-7 vs. Unvaccinated  Oral or intravenous AB, types N/A | Proportions of infants receiving antibiotics  Risk ratio 1.42 (0.70 – 2.88) | Very low |
| Van Werkhoven 2021  (26) | Netherlands  WHO EUR  2008 – 2010, follow up period until 2013 | Adults ≥65 years  N = 40 426  Antibiotic data collection: GP database | PCV 13 vs. Placebo  Doxycycline antibiotics, penicillins, B-lactam inhibitor combinations | Number of antibiotic prescriptions  LRTI-related: VE 4.2% (-1.0% to 9.1%), P = 0.109  Total: VE -0.4% (-4.9% to 3.9%), P = 0.859 | High |
| Veenhoven 2003  (27) | Netherlands  WHO EUR  1998 – 2002 | Children with previous AOM aged 1 – 7 years  N = 383  Antibiotic data collection: parental self-report | PCV7 vs. Hepatitis A or B Vaccine  AB types N/A | Days of ear-related antibiotic use  Reported no significant difference | Very low |
| Yilmaz 2013  (28) | N/A | Adults with COPD  N = 144  Antibiotic data collection: N/A | PPV23 vs. Placebo  AB types N/A | Number of antibiotic courses    Risk ratio 0.24 (0.12 – 0.50) | Very low |
| **Influenza plus pneumococcal vaccine** | | | | | |
| Jansen 2008  (29) | Netherlands  WHO EUR  2003 – 2005 | Children with previously diagnosed RTIs aged 18 – 72 months  N = 579  Antibiotic data collection: parental self-report and clinician records | Trivalent Influenza Vaccine vs. PCV7  Trivalent Influenza Vaccine vs. Recombinant HBV vaccine + placebo  AB types N/A | Number of antibiotic prescriptions  Influenza: Incidence rate ratio 0.89 (0.50 – 1.61)  TIV+PCV: Incidence rate ratio 0.73 (0.40 –1.32), P = 0.57 | Low (Influenza)    Moderate (PCV) |

**Observational studies:**

| Study ID | Country, WHO region, data period | Population, N, data source | Vaccine type,  Antibiotic type | Outcome,  Estimate of Effect (95% CI) | Study quality |
| --- | --- | --- | --- | --- | --- |
| **Influenza vaccine** | | | | | |
| Baquero Ubeda 2010  (30) | Spain  WHO EUR  1993, 1995, 1997, 2000, 2003 | Adults aged ≥16 years  N = 6,400 (1995 and 1997) and  21,000 (1993, 2001, 2003)  Antibiotic data collection: National Health Survey | Influenza vaccine type N/A  AB types N/A | Proportions of people reporting AB use  Similar AB consumption in  vaccinated (3.86% to 7.74%) and  unvaccinated people (3.09% to 7.0%). Statistically significant for 2003 (p = 0.005). | Critical |
| Bowles 2002  (31) | Canada  WHO RAM  1999 – 2000 | Long term care residents aged 39 – 105 years (median age 84 years)  N = 993  Antibiotic data collection: medical records | Influenza vaccine type N/A  AB types N/A | Proportions of people receiving antibiotics  No significant difference | Critical |
| Cao 2008  (32) | China  WHO WPR  2006 – 2007 | Outpatients with ILI, mean age 33 ± 13 years  N = 476  Antibiotic data collection: clinical records & self-report | Influenza vaccine type N/A  Levofloxacin, cephalosporins,  clindamycin, macrolides | Number of people receiving antibiotics  8 (72.7%) out of 11 vaccinated patients received AB during influenza treatment, while 124/186 unvaccinated patients received AB (66.6%) | Critical |
| Gasparini 2002  (33) | Italy  WHO EUR  2000 – 2001 | Elderly patients aged >65 years  N = 512  Antibiotic data collection: clinical records | Influenza vaccine type N/A  Ampicillin, Azithromycin,  Cephalosporin, Ciprofloxacin,  Macrolide, Trimethoprim | Number of antibiotic courses or prescriptions  No significant difference | Critical |
| Hara 2002  (34) | Japan  WHO WPR  Jan – Mar 1999 | Patients hospitalized at a long-term care unit, mean age 80.4 ± 10.3 years  N = 237  Antibiotic data collection: clinical records | Influenza vaccine type N/A  Oral and injected AB, types N/A | Number of days of antibiotic use  No significant different oral AB  Mean duration of injected AB was shorter in vaccinated than in unvaccinated patients (2.55±5.55 vs. 7.52±11.2; p < 0.001). | Moderate |
| Hardelid 2018  (35) | United Kingdom  WHO EUR  2013 – 2015 | Children aged 2 – 4 years  N = 33 137  Antibiotic data collection: primary care database | Live-Attenuated Influenza Vaccine  Amoxicillin | Number of antibiotic prescriptions  2013/2014: VE is 12.6% (95% CI: 6.7% - 18.2%)  2014/2015: VE is 14.5% (95% CI: 9.6% - 19.2%) | Moderate |
| Klein 2020  (36) | USA  WHO RAM  2010 – 2017 | General population: pediatrics (0-18 years), adults (19-64 years), elderly (≥65 years)  N = N/A  Antibiotic data collection: database | Influenza vaccine type N/A  Broad-spectrum penicillins, macrolides, cephalosporins, fluoroquinolones, tetracyclines, trimethoprim, narrow-spectrum penicillins, aminoglycosides | Number of antibiotic prescriptions  A 10% increase in influenza vaccination rates is associate with a 6.5% decrease in antibiotic use (14.2/1000; 95% CI 6.0 - 22.4; p = .001) | Serious |
| Kwong 2009  (37) | Canada  WHO RAM  1997 – 2005 | Community (all ages)  N = N/A  Antibiotic data collection: database | Influenza vaccine type N/A  Penicillins, cephalosporins, broad spectrum macrolides, quinolones | Number of antibiotic prescriptions  A decrease of annual AB prescriptions from 17.9 to 6.4 per 1000 (RR, 0.36; 95% CI, 0.26–0.49) | Low |
| Lavallée 2002  (38) | France  WHO EUR  1998 – 2000 | Patients aged ≥60 years  N = 270 (90 cases)  Antibiotic data collection: patient self-report | Influenza vaccine type N/A  AB types N/A | Antibiotic use within last 3 months  No significant association | Critical |
| Maltezou 2013  (39) | Greece  WHO EUR  2012 – 2013 | Mothers (mean age 30 years) and their infants  N = 530  Antibiotic data collection: parental self-report | Influvac by Abbott or Vaxigrip by  Sanofi-Aventis  AB types N/A | Proportion of infants receiving antibiotics  AB prescribed to 15.2% of infants of  unvaccinated mothers, 8.3% of infants of vaccinated mothers (p = 0.014) | Serious |
| Muller-Pebody 2021  (40) | United Kingdom  WHO EUR  2013 – 2016 | Children aged 2 – 3 years  N = N/A  Antibiotic data collection: database | Live-Attenuated Influenza Vaccine  Doxyxycline, Ampicillin, Amoxicillin, Erythomycin and other | Number of antibiotic prescriptions  2.7% reduction RTI antibiotic use (95% CI: 2.1% - 3.4%; p < .0001) | Moderate |
| Mustafa 2003  (41) | Saudi Arabia  WHO EMR  Feb – Mar 2000 | Malaysian pilgrims  N = 1431  Antibiotic data collection: clinical records & self-report | Influenza vaccine type N/A  AB types N/A | Number of antibiotic prescriptions  VE is 66% (95%CI 54 – 75) | Critical |
| Nichol 2008  (42) | USA  WHO RAM  2002 – 2006 | Full time higher education students aged ≥ 18 years  N = 12 975  Antibiotic data collection: self-report | Influenza vaccine type N/A  AB types N/A | Proportion of people using antibiotics  OR is 0.54 (0.32 – 0.90) | Serious |
| Palin 2019  (43) | United Kingdom  WHO EUR  2000 – 2015 | Children aged 0 – 4 years  N = >8 million  Antibiotic data collection: clinical records | Influenza vaccine type N/A  AB types N/A | Number of antibiotic prescriptions  Increased likelihood of receiving an antibiotic prescription by 3% (sinusitis) to 17% (LRTIs) | Low |
| Qureshi 2000  (44) | Saudi Arabia  WHO EMR  Feb – Mar 1999 | Pakistani pilgrims, median age 42 ± 50 years  N = 2070  Antibiotic data collection: self-report | Vaxigrip influenza vaccine (Aventis-Pasteur)  AB types N/A | Proportion of people using antibiotics  VE is 41% | Serious |
| Rockenschaub 2020  (45) | United Kingdom  WHO EUR  2015 | Patients with COPD aged 35 – 110 years  N = 19 594  Antibiotic data collection: database | Influenza vaccine type N/A  First-line AB (amoxicillin, doxycycline, clarithromycin), second-line AB (co-amoxiclav, co-trimoxazole, levofloxacin) or prophylactics (azithromycin) | Proportion of antibiotic prescriptions  RR 1.23 (95% CI: 1.17 - 1.29) | Moderate |
| Rodgers 2021  (46) | United Kingdom  WHO EUR  2000 – 2001 | Elderly aged ≥65 years  N = 88 519  Antibiotic data collection: clinical records | Influenza vaccine type N/A  Amoxicillin | Number of antibiotic prescriptions  Hazard Ratio 0.86 (95% CI: 0.81 - 0.92) | Low |
| Salleras 2006  (47) | Spain  WHO EUR  2004 – 2005 | Healthy children aged 3 – 14 years  N = 1951  Antibiotic data collection: parental self-report | Inflexal® V Berna (virosomal  subunit inactivated influenza vaccine)  AB types N/A | Number of antibiotic consumptions  VE is 18.6% (95% CI −4.2 to 36.4) | Moderate |
| Vinograd 2013  (48) | Israel  WHO EUR  2010 – 2011 | Adult cancer patients aged >18 years  N = 849  Antibiotic data collection: clinical records | Seasonal influenza vaccine 2011  AB types N/A | Proportion of people receiving antibiotics  No significant difference | Critical |
| **Pneumococcal vaccine** | | | | | |
| Alari 2016  (49) | France  WHO EUR  2001 – 2014 | Whole population  N = N/A (population data)  Antibiotic data collection: ECDC register data | PCV7, PCV13 or PPV23  Beta-lactams, macrolides | Antibiotic consumption (Defined Daily Doses (DDD) per 1000 inhabitants per day)  Sharp decline of AB consumption attributed to national campaign to promote better-targeted AB use (21.23 DDD/1,000/day 2001-2003 to 16.27 DDD/1,000/day in 2004). Consumption increased to 20.11 DDD/1,000/day in 2014. | High |
| Alleman 2017  (50) | Switzerland  WHO EUR  2004 – 2015 | Outpatients of any age with AOM  N = 1588 (pre-PCV7), 961 (PCV7) and 750 (PCV13)  Antibiotic data collection: clinician report | PCV7 or PCV13  AB types N/A | Proportion of people receiving antibiotics  AB use prior to sampling was reported in 14.8% of cases prior to  PCV7 introduction, in 16.4% in the PCV7 period, and in 14.4% in the PCV13 period | Low |
| Block 2004  (51) | USA  WHO RAM  1992 – 1998  2000 – 2003 | Children with AOM aged 7 – 24 months  N = 419  Antibiotic data collection: clinical records | PCV7  AB types N/A | Proportion of people receiving antibiotics  Fewer children in the pre-PCV7 cohort received AB prior to sampling than in the post-PCV7 cohort (59% versus 76%; P=0.036) | High |
| Bruden 2005  (52) | USA  WHO RAM  1998 – 2002 | Adults (mean age 22.8 to 28.4 years)  N = 1956 (1998), 1873 (1999), 2114 (2000), 1696 (2001), 1305 (2002)  Antibiotic data collection: clinical records | PCV7  AB types N/A | Mean number of antibiotic courses and proportion of people receiving antibiotics  Mean number of AB courses (Oct-Mar) fell steadily from 1.02  in 1998 to 0.61 in 2002 (much reduction occurred prior  to PCV7 introduction in 2001).  The proportion of participants with ≥ 1 AB course fell from 49% in 1998 to 42% in 2000. | High |
| Carstairs 2007  (53) | USA  WHO RAM  2000 - 2002 | Children aged 0 – 2 years  N = 1383  Antibiotic data collection: clinical records | PCV7  AB types N/A | Proportion of children receiving antibiotics  38.4% vaccinated and 34.0% unvaccinated children treated with antibiotics | Critical |
| Choi 2011  (54) | USA  WHO RAM  1996 – 2009 | Children with acute mastoiditis (mean age 44.6 to 50.7 months)  N = 96  Antibiotic data collection: clinical records | PCV7  Penicillin, oxacillin, extended-spectrum penicillin, ceftriaxone,  aminoglycosides, vancomycin,  clindamycin | Proportion of children receiving antibiotics  No significant difference | High |
| Cohen 2006  (55) | France  WHO EUR  2001 – 2004 | Children with AOM aged 6-24 months  N = 1896  Antibiotic data collection: clinical records | PCV 7  AB types N/A | Number of children receiving antibiotics  46.4% vaccinated and 45.7% unvaccinated children received AB 90 days before sampling | Critical |
| Daana 2015  (56) | Israel  WHO EUR  2009 – 2011 | Children aged <6 years  N = 2750  Antibiotic data collection: clinical records and parental self-report | PCV 7  AB types N/A | Proportion of children receiving antibiotics  In 2009, 53.7% received AB (2.6% vaccinated) and in 2011 60.8% received AB prior to sampling (70.4% vaccinated) | Low |
| Daniel 2013  (57) | Australia  WHO WPR  2000 – 2010 | Children with acute mastoiditis (mean age 39 months)  N = 84  Antibiotic data collection: clinical records | PCV7  Oral and injected AB, types N/A | Proportion of children receiving antibiotics  Mean number of days of AB use  Receipt of AB prior to admission increased from 54.8% pre-PCV7 to 77.8%. Mean duration of IV AB reduced from 6.0 to 4.2 days, while overall duration of AB treatment remained static (16.6 days – 17.0  days). | High |
| Danino 2021  (58) | Israel  WHO EUR  2005 – 2018 | Children aged <5 years  N = N/A  Antibiotic data collection: database | PCV7 and PCV13  Amoxicillin, azithromycin, OSGCs and other | Number of antibiotic prescriptions  Overall reduction in dispensed antibiotic prescription rates per 1000 was estimated between aIRR (344.7 [370.9–358.4]) and rIRR (110.4 [96.9–123.7]). | Moderate |
| De Bont 2012  (59) | Netherlands  WHO EUR  2000 – 2010 | Children aged ≤12 years  N = 108 555  Antibiotic data collection: clinical records | PCV7  Oral and topical AB, types N/A | Number of antibiotic prescriptions  A significant increase of 4.9%  (p<0.001) in prescription rates prior to PCV7. An average decline of 3.3% (p<0.001) after PCV7 introduction. | High |
| Dunais 2016  (60) | France  WHO EUR  1999, 2002, 2004, 2006, 2008, 2012 | Children aged 3 – 40 months  N = 1969  Antibiotic data collection: clinical records | PCV7 and PCV13  AB types N/A | Proportion of children receiving antibiotics  After introduction of a nationwide campaign to promote prudent AB use and PCV7 vaccination, AB use was 48.0%, 30.9% & 37.9% in 2004, 2006 & 2008 respectively. AB use after PCV13 introduction was 40.2% in 2012. | High |
| El Turki 2010  (61) | United Kingdom  WHO EUR  2001 - 2008 | Children aged 0 – 18 years  N = 35 184  Antibiotic data collection: database | PCV7  AB types N/A | Number of AB prescriptions per person year  AB prescribing for AOM treatment declined from 54 per 1000 person-year (95% CI: 53–55) in 2001 to 31 per 1000 person-year (95% CI: 30–  31) in 2008. | High |
| El Turki 2011  (62) | United Kingdom  WHO EUR  2002 - 2009 | Children aged 0 – 18 years with pneumonia  N = 813  Antibiotic data collection: database | PCV7  AB types N/A | Number of AB prescriptions per person year  Reduction in pneumonia-related AB treatment among children  <2 years from 1.3/1000 person-years  (95% CI 0.7 to 1.9) in 2007 to 0.3/1000 person years (95% CI 0.01 to 0.6) in 2009. | High |
| Ercan 2011  (63) | Turkey  WHO EUR  2007 – 2008 | Children aged 12 – 59 months  N = 247  Antibiotic data collection: parental self-report | PCV7 Prevenar® (Wyeth)  AB types N/A | Proportion of children receiving antibiotics  No significant difference | Critical |
| Esposito 2007  (64) | Italy  WHO EUR  2002 – 2005 | Children aged 2 – 29 months  N = 1555  Antibiotic data collection: parental self-report | PCV 7  AB types N/A | Number of antibiotic courses  Relative Risk 0.89 (95% CI 0.83–0.94); p = 0.0001 | Moderate |
| Eythorsson 2018  (65) | Iceland  WHO EUR  2005 – 2016 | Children aged <3 years  N = 50 570  Antibiotic data collection: database | PHiD-CV10  First-line penicillins (amoxicillin, phenoxymethylpenicillin), Second-line penicillins (amoxicillin and enzyme inhibitor), First-generation macrolide (erythromycin), Second-generation macrolide (azithromycin, clarithromycin), Cephalosporin and others | Number of antimicrobial prescriptions  The incidence rate was significantly lower in vaccine-eligible compared to non-eligible children: 144.5 and 157.2 prescriptions per 100 person-years respectively (IRR 0.92, 95%CI 0.91–0.93). The vaccine impact against all-cause antimicrobial prescriptions was 5.8% (95%CI 1.6–9.8%). | Moderate |
| Fortanier 2018  (66) | Netherlands  WHO EUR  2006 – 2013 | Children aged <2 years  N = 119 078  Antibiotic data collection: database | PCV7 or PCV10  Amoxicillin, axithromycin and other | Number of antibiotic prescriptions  Switching from PCV7 to PCV10 was associated with a modest 1.6% reduction in outpatient antibiotic use. | Moderate |
| Frazao 2005  (67) | Portugal  WHO EUR  2001 – 2003 | Children aged 6 months – 6 years  N = 695  Antibiotic data collection: parental self-report | PCV7 Prevenar (Wyeth Lederle)  AB types N/A | Proportion of children receiving antibiotics  4% vaccinated and 5% unvaccinated children used AB at time of sampling and 17% and 11% vaccinated vs. unvaccinated in month before sampling | Critical |
| Garbutt 2006  (68) | USA  WHO RAM  2000, 2001, 2003, 2004 | Children aged 0-6 years  N = 327  Antibiotic data collection: clinical records | PCV7  AB types N/A | Proportion of children receiving antibiotics  No differences | High |
| Garcia-Vidal 2012  (69) | Spain  WHO EUR  2006 – 2011 | Adults aged 24 – 84 years  N = 63  Antibiotic data collection: patient self-report | PPV23  AB types N/A | Proportion of people receiving antibiotics  Vaccinated patients received more AB treatments in 4 weeks prior to  treatment for bacteremia than other patients (53.3 vs. 19.4%; p = 0.022) | Critical |
| Gefenaite 2014  (70) | Netherlands  WHO EUR  2002 – 2013 | Children aged 1 – 9 years  N = N/A (population data)  Antibiotic data collection: database | PCV7 and PCV10  Amoxicillin, azithromycin, sulfamethoxazole, trimethoprim | Slight decrease in respiratory AB  prescriptions after the introduction of PCV7 and PCV10. Statistically significant reduction after PCV7 in children aged 3 and 4: −4.94% (95% CI: −5.26 to −4.63) and −9.02% (95% CI: −14.82 to −2.83), respectively. | Low |
| Grivea 2010  (71) | Greece  WHO EUR  2005 – 2007 | Children aged 13 – 76 months  N = 1756  Antibiotic data collection: parental self-report | PCV 7  AB types N/A | Proportion of children receiving antibiotics  AB received during 3 months prior to  surveys by 51.9% of vaccinated and 54.6% of unvaccinated children | Critical |
| Hoang 2019  (72) | France  WHO EUR  2015 | Adults aged 24 – 81 years  N = 119  Antibiotic data collection: self-report | PCV13  B-lactams, macrolides | Number of antibiotic treatments  Vaccinated pilgrims were seven time less likely to present S. pneumoniae carriage post-Hajj compared to those not vaccinated (3.2% vs. 18.0%, OR = 0.15; 95% CI [0.03–0.74], p = 0.02). | Serious |
| Howitz 2017  (73) | Denmark  WHO EUR  2000 – 2014 | Children aged 0 – 15 years  N = N/A (population data)  Antibiotic data collection: database | PCV7 and PCV13  AB types N/A | Incidence rate of antibiotic use (DDD per person/year)  The age adjusted annual  incidence rate of AB use reduced  from 2.9 to 2.5 after PCV7 and  there was a temporal increase after PCV13 introduction, peaking at 3.1 in 2011. | High |
| Johansson Kostenniemi 2018  (74) | Sweden  WHO EUR  2005 – 2014 | Whole population (children, adults and elderly)  N = 259 183  Antibiotic data collection: database | PCV7 or PCV13  AB types N/A | Number of antibiotic prescriptions per 1000 persons/year  Antibiotic consumption for URTI decreased by 37.1%, with the largest decrease occurring in children aged 0–4 years. For pneumonia, the incidence significantly decreased by 28.6% for children aged 0–4 years, with no significant changes in older children or adults. | Moderate |
| Kinlaw 2017  (75) | Denmark  WHO EUR  2004 – 2013 | Children aged 0-1 years (birth cohort)  N = 561 729  Antibiotic data collection: database | PCV7 and PCV13  AB types N/A | 1-year risk (%) of having ≥ 1 AB  prescription  The overall 1-year risk of having at least 1 redeemed AB prescription decreased from 40.7% amongst births in 2004 to 34.6% in births in 2012. | Low |
| Lakshman 2003  (76) | United Kingdom  WHO EUR  2000 – 2001 | Children aged 2 – 5 years  N = 276 (summer) and 331 (winter)  Antibiotic data collection: N/A | PCV 7  AB types N/A | Proportion of children using antibiotics  No significant difference | Critical |
| Lau 2015  (77) | United Kingdom  WHO EUR  2002 – 2012 | Children <10 years  N = 567 275  Antibiotic data collection: database | PCV7 and PCV13  AB types N/A | Number of antibiotic prescriptions per 1000 person years  18.9% (95% CI, 16.0-21.7%) monthly reduction in AB prescription rates overall after PCV7 introduction. Replacement of PCV7 with PCV13  was associated with a further 12.2% (95%CI, 8.6-15.6%) monthly reduction (only significant for 5-9 year-olds (18.6%; 95%CI, 13.1-23.8%)). | Low |
| Lee 2013  (78) | Taiwan  WHO WPR  2009 – 2012 | Children (mean age 3.3 years)  N = 11  Antibiotic data collection: clinical records | PCV7 or PCV13  Vancomycin, cefotaxime, ampicillin,  sulbactam, ceftriaxone, azithromycin | Antibiotic regimens  All vaccinated and unvaccinated children were treated with antibiotics | Critical |
| Lewnard 2020  (79) | Afghanistan, Angola, Armenia, Burundi, Ethiopia, Haiti, Lao People’s Democratic Republic, Malawi, Nepal, Pakistan, the Philippines, Senegal, Sierra Leone, South Africa, Tajikistan, the United Republic of Tanzania, Uganda and Zimbabwe  All WHO regions  2006 – 2018 | Children aged <5 years  N = 65 815  Antibiotic data collection: database (incl. parental self-report data) | PCV10 or PCV13  AB types N/A | Number of children using antibiotics  PCV10/13 reduce antibiotic consumption and it is estimated that pneumococcal conjugate vaccines confer 19.7% (95% confidence interval, 3.4–43.4%) protection against antibiotic-treated episodes of acute respiratory infection and diarrhea. Pneumococcal and vaccines prevent 23.8 million episodes of antibiotic-treated illness among children <5 years in LMICs each year. | Moderate |
| Mackenzie 2009  (80) | Australia  WHO WPR  1996 - 2006 | Infants aged <2 years  N = 135  Antibiotic data collection: N/A | PCV7  AB types N/A | Mean number of AB prescriptions  Vaccinated infants received  fewer AB prescriptions (mean 3.54) than unvaccinated infants (mean 4.51): Mean difference -0.97 (95% CI -2.30 – 0.37) | Critical |
| McEllistrem 2005  (81) | USA  WHO RAM  1999 - 2002 | Children with AOM  N = 488  Antibiotic data collection: self-report | PCV7  AB types N/A | Proportion of children receiving antibiotics  Little differences: 65% pre-PCV7 in 1999, 65% post PCV7 in 2000/2001 and 56% in 2002. | High |
| Mintegi 2006  (82) | Spain  WHO EUR  2004 - 2005 | Children aged 6 – 24 months  N = 770  Antibiotic data collection: clinical records | PCV7  Intramuscular ceftriaxone | Proportion of children receiving antibiotics  1.3% fully vaccinated (n=215), 1.7% incompletely vaccinated (n=175), and 7.1% unvaccinated children (n=380) received AB treatment | Critical |
| Mykietiuk 2006  (83) | Spain  WHO EUR  1995 – 2004 | Adults aged 48 – 87 years  N = 499  Antibiotic data collection: clinical records | PPV23  AB types N/A | Proportion of people receiving antibiotics  No significant difference | Critical |
| Nunes 2016  (84) | Portugal  WHO EUR  1996 – 1999, 2001 – 2003, 2006 – 2007, 2009 – 2010 | Children aged 0-6 years  N = 7551  Antibiotic data collection: parental self-report | PCV7, PCV10 and PCV13  AB types N/A | Proportion of children taking antibiotics  Antimicrobial use decreased over the years (p < 0.001) from 8.3% in 1996 to 4.9% in 2010. | High |
| Ouldali 2019  (85) | France  WHO EUR  2001 – 2018 | Children with AOM aged 6 – 24 months  N = 10 204  Antibiotic data collection: clinical records | PCV7 and PCV13  Amoxicillin, cephalosporins, macrolides, penicillins | Proportion of children using antibiotics  Global antibiotic use decreased over the study period (−22.2%; 95% CI −33.0 to −11.3), but aminopenicillin use remained high. | Moderate |
| Palmu 2018  (86) | Finland  WHO EUR  2004 – 2014 | Children aged 0 – 4 years  N = 497 382 (pre-PCV10) and  512 343 (post-PCV10)  Antibiotic data collection: database | PCV10  AB types N/A | Incidence rate of antibiotic purchases  Relative rate reduction was 17.5% (95% CI: 17.0–18.1) and the absolute rate reduction 0.20 per person-year post-PCV10 compared with pre-  PCV10. | High |
| Ribitzky-Eisner 2016  (87) | Israel  WHO EUR  2005 – 2012 | Children aged 3 – 36 months diagnosed with occult bacteraemia  N = 89  Antibiotic data collection: clinical records | PCV13 (Prevenar 13)  Oral amoxicillin, intramuscular/intravenous ceftriaxone | Proportion of children receiving antibiotics  No significant difference | High |
| Roddy 2007  (88) | USA  WHO RAM  1995 – 2005 | Patients with mastoiditis aged ≤ 19 years  N = 122  Antibiotic data collection: clinical records and database | PCV7  AB types N/A | Proportion of people using antibiotics  Almost identical rates of AB use  reported for pre-/post-  PCV (47% and 48% respectively). | High |
| Shapiro 2011  (89) | USA  WHO RAM  1998 – 2007 | Children aged <18 years  with acute sinusitis  N = 389  Antibiotic data collection: database | PCV7  Cephalosporins, macrolides, amoxicillin clavulanate, amoxicillin,  quinolones, sulfonamides | Proportion of acute sinusitis visits in which antibiotics were prescribed  No change in the overall rate at  which AB were prescribed (P = .71).  However, there was an increase in the use of firstline  amoxicillin over time from 19% (95% CI: 10%–35%) to 58% (95% CI: 40%–74%) during the study (P = .01). | High |
| Stevens 2013  (90) | USA  WHO RAM  1992 – 2009 | People aged ≥5 years old  N = N/A (population data)  Antibiotic data collection: database | PCV7  Penicillins, cephalosporins,  macrolides, tetracyclines or trimethoprim/sulfamethoxazole | Number of antibiotics prescriptions per 1000 population/year  Antimicrobial prescribing increased by 59% from 285 prescriptions per 1000 persons per year to 454 prescriptions per 1000 persons per year between 1992 and 2009 (p<0.001). | High |
| Stolbrink 2019  (91) | United Kingdom  WHO EUR  2010 – 2015 | Patients with COPD aged 70.83 ± 10.9 years  N = 22 003  Antibiotic data collection: database | Pneumococcal vaccine (any)  Doxycycline, ertyhromycin/clarithromycin, amoxicillin | Proportion of people receiving antibiotics  Prior pneumoccocal vaccination was associated with repeat antibiotic prescriptions in COPD patients: OR 1.33, 95% CI 1.14 - 1.55. | Moderate |
| Tamir 2015  (92) | Israel  WHO EUR  2008 – 2013 | Children aged <6 years  N = 279  Antibiotic data collection: clinical records | PCV7 or PCV13  AB types N/A | Proportion of children using antibiotics  Fewer PCV7 vaccinated children had a recent history of AB use (30%) compared with unvaccinated children (48%; p = 0.04). Compared with PCV7 vaccinated children, more children vaccinated with PCV13 had a recent history of AB use (65%; p = 0.03) | Critical |
| Tief 2016  (93) | Germany  WHO EUR  2010 – 2013 | Children aged 0 – 18 years  N = 2569  Antibiotic data collection: clinical records | Pneumococcal vaccine (any)  AB types N/A | Antibiotic prescriptions  Low Kullback-Leibler divergence estimate of difference of KL=0.008 between vaccinated and  unvaccinated children | Critical |
| Vasoo 2011  (94) | Singapore  WHO WPR  1998, 2007 – 2008 | Children (age N/A)  N = 813  Antibiotic data collection: N/A | PCV7  AB types N/A | Proportion of children using antibiotics  Little difference in AB use between pre-PCV7 (35.8%) and post-PCV7  (38.8%). | High |
| Waddle 2009  (95) | USA  WHO RAM  1997 – 1999, 2001 – 2004 | Children aged 3 – 36 months  N = 3279  Antibiotic data collection: N/A | PCV7  AB types N/A | Proportion of children receiving antibiotics  In the pre-PCV7 era, 60.8% of patients received AB and in the post-PCV7 era, 57.2% received AB. | High |
| Zhou 2008  (96) | USA  WHO RAM  1997 – 2004 | Children aged <2 years  N = 20 628 to 153 812  Antibiotic data collection: medical records | PCV7  Cephalosporins, erythromycin and  macrolides, sulfonamides and  combinations | Number of prescriptions per  1000 person years/year  Decrease of 41.9% from 1244 AB prescriptions for AOM per 1000  children to 722 prescriptions per 1000 children (P< .001). Differs per AB type. | High |
| **Influenza plus pneumococcal vaccine** | | | | | |
| Dohna-Schwake 2008  (97) | Germany  WHO EUR  Data period N/A | Children with Neuromuscular Disorders  N = 34  Antibiotic data collection: parental self-report | Influenza and pneumococcal vaccine (any)  AB types N/A | Mean number of antibiotic courses  No statistical significance | Critical |
| Mahamat 2013  (98) | France  WHO EUR  2003 – 2005 | Elderly aged >65 years  N = 46 477  Antibiotic data collection: database | Seasonal influenza vaccine and PPV23  Penicillins or penicillin combinations,  macrolides, fluoroquinolones,  cephalosporins | Antibiotic consumption before and after vaccination campaign  No significant difference | Serious |

**References:**

1. Allsup S, Gosney M, Haycox A, Regan M. Cost-benefit evaluation of routine influenza immunisation in people 65-74 years of age. Health Technol Assess. 2003;7(24):iii-x, 1-65.

2. Belshe RB, Mendelman PM, Treanor J, King J, Gruber WC, Piedra P, et al. The efficacy of live attenuated, cold-adapted, trivalent, intranasal influenzavirus vaccine in children. N Engl J Med. 1998;338(20):1405-12.

3. Bridges CB, Thompson WW, Meltzer MI, Reeve GR, Talamonti WJ, Cox NJ, et al. Effectiveness and Cost-Benefit of Influenza Vaccination of Healthy Working AdultsA Randomized Controlled Trial. JAMA. 2000;284(13):1655-63.

4. Dbaibo G, Amanullah A, Claeys C, Izu A, Jain VK, Kosalaraksa P, et al. Quadrivalent Influenza Vaccine Prevents Illness and Reduces Healthcare Utilization Across Diverse Geographic Regions During Five Influenza Seasons: A Randomized Clinical Trial. The Pediatric Infectious Disease Journal. 2020;39(1):e1-e10.

5. Esposito S, Marchisio P, Cavagna R, Gironi S, Bosis S, Lambertini L, et al. Effectiveness of influenza vaccination of children with recurrent respiratory tract infections in reducing respiratory-related morbidity within the households. Vaccine. 2003;21(23):3162-8.

6. Gao ZC, Li JT, Zhan S. Preventive and curative effects of Card Shu Ning combined with influenza vaccine on senile chronic bronchitis complicated with acute infection. Chinese journal of biologicals. 2011;24(10):1214‐6.

7. Hoberman A, Greenberg DP, Paradise JL, Rockette HE, Lave JR, Kearney DH, et al. Effectiveness of Inactivated Influenza Vaccine in Preventing Acute Otitis Media in Young ChildrenA Randomized Controlled Trial. JAMA. 2003;290(12):1608-16.

8. Hurwitz ES, Haber M, Chang A, Shope T, Teo S, Ginsberg M, et al. Effectiveness of Influenza Vaccination of Day Care Children in Reducing Influenza-Related Morbidity Among Household Contacts. JAMA. 2000;284(13):1677-82.

9. Loeb M, Russell ML, Moss L, Fonseca K, Fox J, Earn DJD, et al. Effect of Influenza Vaccination of Children on Infection Rates in Hutterite Communities: A Randomized Trial. JAMA. 2010;303(10):943-50.

10. Marchisio P, Cavagna R, Maspes B, Gironi S, Esposito S, Lambertini L, et al. Efficacy of Intranasal Virosomal Influenza Vaccine in the Prevention of Recurrent Acute Otitis Media in Children. Clinical Infectious Diseases. 2002;35(2):168-74.

11. Marchisio P, Esposito S, Bianchini S, Dusi E, Fusi M, Nazzari E, et al. Efficacy of Injectable Trivalent Virosomal-Adjuvanted Inactivated Influenza Vaccine in Preventing Acute Otitis Media in Children With Recurrent Complicated or Noncomplicated Acute Otitis Media. The Pediatric Infectious Disease Journal. 2009;28(10):855-9.

12. Nichol KL, Mendelman PM, Mallon KP, Jackson LA, Gorse GJ, Belshe RB, et al. Effectiveness of Live, Attenuated Intranasal Influenza Virus Vaccine in Healthy, Working Adults: A Randomized Controlled Trial. JAMA. 1999;282(2):137-44.

13. Pepin S, Samson SI, Alvarez FP, Dupuy M, Gresset-Bourgeois V, De Bruijn I. Impact of a quadrivalent inactivated influenza vaccine on influenza-associated complications and health care use in children aged 6 to 35 months: Analysis of data from a phase III trial in the Northern and Southern Hemispheres. Vaccine. 2019;37(13):1885-8.

14. Pisu M, Meltzer MI, Hurwitz ES, Haber M. Household-based costs and benefits of vaccinating healthy children in daycare against influenza virus. PharmacoEconomics. 2005;23(1):55-67.

15. PRINCIPI N, ESPOSITO S, MARCHISIO P, GASPARINI R, CROVARI P. Socioeconomic impact of influenza on healthy children and their families. The Pediatric Infectious Disease Journal. 2003;22(10):S207-S10.

16. Vesikari T, Fleming DM, Aristegui JF, Vertruyen A, Ashkenazi S, Rappaport R, et al. Safety, efficacy, and effectiveness of cold-adapted influenza vaccine-trivalent against community-acquired, culture-confirmed influenza in young children attending day care. Pediatrics. 2006;118(6):2298-312.

17. Dagan R, Sikuler-Cohen M, Zamir O, Janco J, Givon-Lavi N, Fraser D. Effect of a conjugate pneumococcal vaccine on the occurrence of respiratory infections and antibiotic use in day-care center attendees. The Pediatric Infectious Disease Journal. 2001;20(10):951-8.

18. FIREMAN B, BLACK SB, SHINEFIELD HR, LEE J, LEWIS E, RAY P. Impact of the pneumococcal conjugate vaccine on otitis media. The Pediatric Infectious Disease Journal. 2003;22(1):10-6.

19. Karppinen S, Toivonen L, Schuez-Havupalo L, Teros-Jaakkola T, Waris M, Auranen K, et al. Effectiveness of the ten-valent pneumococcal Haemophilus influenzae protein D conjugate vaccine (PHiD-CV10) against all respiratory tract infections in children under two years of age. Vaccine. 2019;37(22):2935-41.

20. Nieminen H, Rinta-Kokko H, Jokinen J, Puumalainen T, Moreira M, Borys D, et al. Effectiveness of the 10-valent pneumococcal conjugate vaccine among girls, boys, preterm and low-birth-weight infants – Results from a randomized, double-blind vaccine trial. Vaccine. 2019;37(28):3715-21.

21. O'Grady KF, Chang AB, Cripps A, Mulholland EK, Smith-Vaughan H, Wood N, et al. The clinical, immunological and microbiological impact of the 10-valent pneumococcal-Protein D conjugate vaccine in children with recurrent protracted bacterial bronchitis, chronic suppurative lung disease and bronchiectasis: A multi-centre, double-blind, randomised controlled trial. Hum Vaccin Immunother. 2018;14(11):2768-79.

22. Palmu AA, Jokinen J, Nieminen H, Rinta-Kokko H, Ruokokoski E, Puumalainen T, et al. Effect of pneumococcal Haemophilus influenzae protein D conjugate vaccine (PHiD-CV10) on outpatient antimicrobial purchases: a double-blind, cluster randomised phase 3-4 trial. Lancet Infect Dis. 2014;14(3):205-12.

23. Palmu AA, Jokinen J, Nieminen H, Rinta-Kokko H, Ruokokoski E, Puumalainen T, et al. Vaccine-preventable disease incidence of pneumococcal conjugate vaccine in the Finnish invasive pneumococcal disease vaccine trial. Vaccine. 2018;36(14):1816-22.

24. Steentoft J, Konradsen HB, Hilskov J, Gislason G, Andersen JR. Response to pneumococcal vaccine in chronic obstructive lung disease—The effect of ongoing, systemic steroid treatment. Vaccine. 2006;24(9):1408-12.

25. van Gils EJM, Veenhoven RH, Hak E, Rodenburg GD, Bogaert D, IJzerman EPF, et al. Effect of Reduced-Dose Schedules With 7-Valent Pneumococcal Conjugate Vaccine on Nasopharyngeal Pneumococcal Carriage in Children: A Randomized Controlled Trial. JAMA. 2009;302(2):159-67.

26. van Werkhoven CH, Bolkenbaas M, Huijts SM, Verheij TJM, Bonten MJM. Effects of 13-valent pneumococcal conjugate vaccination of adults on lower respiratory tract infections and antibiotic use in primary care: secondary analysis of a double-blind randomized placebo-controlled study. Clin Microbiol Infect. 2021;27(7):995-9.

27. Veenhoven R, Bogaert D, Uiterwaal C, Brouwer C, Kiezebrink H, Bruin J, et al. Effect of conjugate pneumococcal vaccine followed by polysaccharide pneumococcal vaccine on recurrent acute otitis media: a randomised study. Lancet. 2003;361(9376):2189-95.

28. Yilmaz D, Uzaslan E, Ege E. Impact Of Pneumococcal Polysaccharide Vaccine On Acute Exacerbation And Quality Of Life In COPD Patients. A103 CHRONIC OBSTRUCTIVE PULMONARY DISEASE EXACERBATIONS: INFECTIOUS MECHANISMS2013. p. A2182-A.

29. Jansen AG, Sanders EA, Hoes AW, van Loon AM, Hak E. Effects of influenza plus pneumococcal conjugate vaccination versus influenza vaccination alone in preventing respiratory tract infections in children: a randomized, double-blind, placebo-controlled trial. J Pediatr. 2008;153(6):764-70.

30. Baquero JL, Barberán J, Martínez D. [Critical study of spanish adult consumer profile of antibiotics on the basis of National Surveys of Health in 1993, 1995, 1997, 2001 and 2003]. Rev Esp Quimioter. 2010;23(3):126-34.

31. Bowles SK, Lee W, Simor AE, Vearncombe M, Loeb M, Tamblyn S, et al. Use of oseltamivir during influenza outbreaks in Ontario nursing homes, 1999-2000. J Am Geriatr Soc. 2002;50(4):608-16.

32. Cao B, Li R, Liu YM, Cao ZX, Geng XQ, Lau LT, et al. [The impact of antibiotic treatment in patients with influenza-like illness]. Zhonghua Jie He He Hu Xi Za Zhi. 2008;31(7):483-7.

33. Gasparini R, Lucioni C, Lai P, Maggioni P, Sticchi L, Durando P, et al. Cost-benefit evaluation of influenza vaccination in the elderly in the Italian region of Liguria. Vaccine. 2002;20 Suppl 5:B50-4.

34. Hara Y, Hagihara A, Ikematu H, Nobutomo K. Efficacy of influenza vaccine among elderly patients by physical activity status. Environ Health Prev Med. 2002;7(5):183-8.

35. Hardelid P, Ghebremichael-Weldeselassie Y, Whitaker H, Rait G, Gilbert R, Petersen I. Effectiveness of live attenuated influenza vaccine in preventing amoxicillin prescribing in preschool children: a self-controlled case series study. J Antimicrob Chemother. 2018;73(3):779-86.

36. Klein EY, Schueller E, Tseng KK, Morgan DJ, Laxminarayan R, Nandi A. The Impact of Influenza Vaccination on Antibiotic Use in the United States, 2010–2017. Open Forum Infectious Diseases. 2020;7(7).

37. Kwong JC, Maaten S, Upshur RE, Patrick DM, Marra F. The effect of universal influenza immunization on antibiotic prescriptions: an ecological study. Clin Infect Dis. 2009;49(5):750-6.

38. Lavallée P, Perchaud V, Gautier-Bertrand M, Grabli D, Amarenco P. Association between influenza vaccination and reduced risk of brain infarction. Stroke. 2002;33(2):513-8.

39. Maltezou HC, Fotiou A, Antonakopoulos N, Kallogriopoulou C, Katerelos P, Dimopoulou A, et al. Impact of postpartum influenza vaccination of mothers and household contacts in preventing febrile episodes, influenza-like illness, healthcare seeking, and administration of antibiotics in young infants during the 2012-2013 influenza season. Clin Infect Dis. 2013;57(11):1520-6.

40. Muller-Pebody B, Sinnathamby MA, Warburton F, Rooney G, Andrews N, Whitaker H, et al. Impact of the childhood influenza vaccine programme on antibiotic prescribing rates in primary care in England. Vaccine. 2021;39(45):6622-7.

41. Mustafa AN, Gessner BD, Ismail R, Yusoff AF, Abdullah N, Ishak I, et al. A case-control study of influenza vaccine effectiveness among Malaysian pilgrims attending the Haj in Saudi Arabia. Int J Infect Dis. 2003;7(3):210-4.

42. Nichol KL, D'Heilly S, Ehlinger EP. Influenza vaccination among college and university students: impact on influenzalike illness, health care use, and impaired school performance. Arch Pediatr Adolesc Med. 2008;162(12):1113-8.

43. Palin V, Mölter A, Belmonte M, Ashcroft DM, White A, Welfare W, et al. Antibiotic prescribing for common infections in UK general practice: variability and drivers. Journal of Antimicrobial Chemotherapy. 2019;74(8):2440-50.

44. Qureshi H, Gessner BD, Leboulleux D, Hasan H, Alam SE, Moulton LH. The incidence of vaccine preventable influenza-like illness and medication use among Pakistani pilgrims to the Haj in Saudi Arabia. Vaccine. 2000;18(26):2956-62.

45. Rockenschaub P, Jhass A, Freemantle N, Aryee A, Rafiq M, Hayward A, et al. Opportunities to reduce antibiotic prescribing for patients with COPD in primary care: a cohort study using electronic health records from the Clinical Practice Research Datalink (CPRD). Journal of Antimicrobial Chemotherapy. 2019;75(1):243-51.

46. Rodgers LR, Streeter AJ, Lin N, Hamilton W, Henley WE. Impact of influenza vaccination on amoxicillin prescriptions in older adults: A retrospective cohort study using primary care data. PLoS One. 2021;16(1):e0246156.

47. Salleras L, Domínguez A, Pumarola T, Prat A, Marcos MA, Garrido P, et al. Effectiveness of virosomal subunit influenza vaccine in preventing influenza-related illnesses and its social and economic consequences in children aged 3-14 years: a prospective cohort study. Vaccine. 2006;24(44-46):6638-42.

48. Vinograd I, Eliakim-Raz N, Farbman L, Baslo R, Taha A, Sakhnini A, et al. Clinical effectiveness of seasonal influenza vaccine among adult cancer patients. Cancer. 2013;119(22):4028-35.

49. Alari A, Chaussade H, Domenech De Cellès M, Le Fouler L, Varon E, Opatowski L, et al. Impact of pneumococcal conjugate vaccines on pneumococcal meningitis cases in France between 2001 and 2014: a time series analysis. BMC Med. 2016;14(1):211.

50. Allemann A, Frey PM, Brugger SD, Hilty M. Pneumococcal carriage and serotype variation before and after introduction of pneumococcal conjugate vaccines in patients with acute otitis media in Switzerland. Vaccine. 2017;35(15):1946-53.

51. Block SL, Hedrick J, Harrison CJ, Tyler R, Smith A, Findlay R, et al. Community-wide vaccination with the heptavalent pneumococcal conjugate significantly alters the microbiology of acute otitis media. Pediatr Infect Dis J. 2004;23(9):829-33.

52. Bruden DL, Hennessy TW, Butler JC, Hurlburt DA, Parks DJ, Bulkow LR. Evaluation of a volunteer sample in nasopharyngeal colonization surveys for Streptococcus pneumoniae in rural Alaska. Int J Circumpolar Health. 2005;64(1):16-25.

53. Carstairs KL, Tanen DA, Johnson AS, Kailes SB, Riffenburgh RH. Pneumococcal bacteremia in febrile infants presenting to the emergency department before and after the introduction of the heptavalent pneumococcal vaccine. Ann Emerg Med. 2007;49(6):772-7.

54. Choi SS, Lander L. Pediatric acute mastoiditis in the post-pneumococcal conjugate vaccine era. Laryngoscope. 2011;121(5):1072-80.

55. Cohen R, Levy C, de La Rocque F, Gelbert N, Wollner A, Fritzell B, et al. Impact of pneumococcal conjugate vaccine and of reduction of antibiotic use on nasopharyngeal carriage of nonsusceptible pneumococci in children with acute otitis media. The Pediatric infectious disease journal. 2006;25(11):1001-7.

56. Daana M, Rahav G, Hamdan A, Thalji A, Jaar F, Abdeen Z, et al. Measuring the effects of pneumococcal conjugate vaccine (PCV7) on Streptococcus pneumoniae carriage and antibiotic resistance: the Palestinian-Israeli Collaborative Research (PICR). Vaccine. 2015;33(8):1021-6.

57. Daniel M, Gautam S, Scrivener TA, Meller C, Levin B, Curotta J. What effect has pneumococcal vaccination had on acute mastoiditis? J Laryngol Otol. 2013;127 Suppl 1:S30-4.

58. Danino D, van der Beek BA, Givon-Lavi N, Greenberg D, Ben-Shimol S, Dagan R. Unraveling the Impact of Pneumococcal Conjugate Vaccines on Ambulatory Antibiotic Drug Consumption in Young Children: An Interrupted Time-Series Analysis. Clinical Infectious Diseases. 2021;73(7):1268-78.

59. de Bont EG, van Loo IH, Dukers-Muijrers NH, Hoebe CJ, Bruggeman CA, Dinant GJ, et al. Oral and topical antibiotic prescriptions for children in general practice. Arch Dis Child. 2013;98(3):228-31.

60. Dunais B, Bruno P, Touboul P, Degand N, Sakarovitch C, Fontas E, et al. Impact of the 13-valent pneumococcal conjugate vaccine on nasopharyngeal carriage of Streptococcus pneumoniae among children attending group daycare in southeastern France. Pediatr Infect Dis J. 2015;34(3):286-8.

61. El Turki AA, Hsia YF, Long PF, Sharland M, Wong ICK. Impact of 7-valent pneumococcal conjugate vaccine (PCV-7) on otitis media and antibiotic prescribing for otitis media on children and adolescent aged 0-18 years in United Kingdom. PharmacoepidemiolDrug Saf. 2010;Conference:(UK and Ireland):648.

62. El Turki AA, Hsia Y, Saxena S, Long P, Wong ICK, Sharland M. Impact of 7-valent pneumococcal conjugate vaccine (PVC-7) on the incidence and treatment of pneumonia diagnosed in primary care in children and adolescents in UK. Arch Dis Child. 2011;96(Suppl 1):A5.

63. Ercan TE, Severge B, Topkaya A, Ercan RG, Altınkaya N. Effect of the pneumococcal conjugate vaccine on pneumococcal carriage in Turkish children. Pediatr Int. 2011;53(2):224-30.

64. Esposito S, Lizioli A, Lastrico A, Begliatti E, Rognoni A, Tagliabue C, et al. Impact on respiratory tract infections of heptavalent pneumococcal conjugate vaccine administered at 3, 5 and 11 months of age. Respir Res. 2007;8(1):12.

65. Eythorsson E, Sigurdsson S, Hrafnkelsson B, Erlendsdóttir H, Haraldsson Á, Kristinsson KG. Impact of the 10-valent pneumococcal conjugate vaccine on antimicrobial prescriptions in young children: a whole population study. BMC Infectious Diseases. 2018;18(1):505.

66. Fortanier AC, Venekamp RP, Stellato RK, Sanders EAM, Damoiseaux RAMJ, Hoes AW, et al. Outpatient antibiotic use in Dutch infants after 10-valent pneumococcal vaccine introduction: a time-series analysis. BMJ Open. 2018;8(6):e020619.

67. Frazão N, Brito-Avô A, Simas C, Saldanha J, Mato R, Nunes S, et al. Effect of the seven-valent conjugate pneumococcal vaccine on carriage and drug resistance of Streptococcus pneumoniae in healthy children attending day-care centers in Lisbon. Pediatr Infect Dis J. 2005;24(3):243-52.

68. Garbutt J, Rosenbloom I, Wu J, Storch GA. Empiric first-line antibiotic treatment of acute otitis in the era of the heptavalent pneumococcal conjugate vaccine. Pediatrics. 2006;117(6):e1087-94.

69. Garcia-Vidal C, Ardanuy C, Gudiol C, Cuervo G, Calatayud L, Bodro M, et al. Clinical and microbiological epidemiology of Streptococcus pneumoniae bacteremia in cancer patients. J Infect. 2012;65(6):521-7.

70. Gefenaite G, Bijlsma MJ, Bos HJ, Hak E. Did introduction of pneumococcal vaccines in the Netherlands decrease the need for respiratory antibiotics in children? Analysis of 2002 to 2013 data. Euro Surveill. 2014;19(44).

71. Grivea IN, Tsantouli AG, Chryssanthopoulou DC, Syrogiannopoulos GA. Interaction of the heptavalent pneumococcal conjugate vaccine and the use of individual antibiotics among children on nasopharyngeal colonization with erythromycin-resistant Streptococcus pneumoniae. Eur J Clin Microbiol Infect Dis. 2010;29(1):97-105.

72. Hoang V-T, Meftah M, Anh Ly TD, Drali T, Yezli S, Alotaibi B, et al. Bacterial respiratory carriage in French Hajj pilgrims and the effect of pneumococcal vaccine and other individual preventive measures: A prospective cohort survey. Travel Medicine and Infectious Disease. 2019;31:101343.

73. Howitz MF, Harboe ZB, Ingels H, Valentiner-Branth P, Mølbak K, Djurhuus BD. A nationwide study on the impact of pneumococcal conjugate vaccination on antibiotic use and ventilation tube insertion in Denmark 2000-2014. Vaccine. 2017;35(43):5858-63.

74. Johansson Kostenniemi U, Palm J, Silfverdal SA. Reductions in otitis and other respiratory tract infections following childhood pneumococcal vaccination. Acta Paediatr. 2018.

75. Kinlaw AC, Stürmer T, Lund JL, Pedersen L, Kappelman MD, Daniels JL, et al. Trends in Antibiotic Use by Birth Season and Birth Year. Pediatrics. 2017;140(3).

76. Lakshman R, Murdoch C, Race G, Burkinshaw R, Shaw L, Finn A. Pneumococcal nasopharyngeal carriage in children following heptavalent pneumococcal conjugate vaccination in infancy. Arch Dis Child. 2003;88(3):211-4.

77. Lau WC, Murray M, El-Turki A, Saxena S, Ladhani S, Long P, et al. Impact of pneumococcal conjugate vaccines on childhood otitis media in the United Kingdom. Vaccine. 2015;33(39):5072-9.

78. Lee MR, Chen CM, Chuang TY, Huang YT, Hsueh PR. Capsular serotypes and antimicrobial susceptibilities of Streptococcus pneumoniae causing invasive pneumococcal disease from 2009-2012 with an emphasis on serotype 19A in bacteraemic pneumonia and empyema and β-lactam resistance. Int J Antimicrob Agents. 2013;42(5):395-402.

79. Lewnard JA, Lo NC, Arinaminpathy N, Frost I, Laxminarayan R. Childhood vaccines and antibiotic use in low- and middle-income countries. Nature. 2020;581(7806):94-9.

80. Mackenzie GA, Carapetis JR, Leach AJ, Morris PS. Pneumococcal vaccination and otitis media in Australian Aboriginal infants: comparison of two birth cohorts before and after introduction of vaccination. BMC Pediatr. 2009;9:14.

81. McEllistrem MC, Adams JM, Patel K, Mendelsohn AB, Kaplan SL, Bradley JS, et al. Acute otitis media due to penicillin-nonsusceptible Streptococcus pneumoniae before and after the introduction of the pneumococcal conjugate vaccine. Clin Infect Dis. 2005;40(12):1738-44.

82. Mintegi S, Benito J, González M, Astobiza E, Sanchez J, Santiago M. Impact of the pneumococcal conjugate vaccine in the management of highly febrile children aged 6 to 24 months in an emergency department. Pediatr Emerg Care. 2006;22(8):566-9.

83. Mykietiuk A, Carratalà J, Domínguez A, Manzur A, Fernández-Sabé N, Dorca J, et al. Effect of prior pneumococcal vaccination on clinical outcome of hospitalized adults with community-acquired pneumococcal pneumonia. Eur J Clin Microbiol Infect Dis. 2006;25(7):457-62.

84. Nunes S, Félix S, Valente C, Simões AS, Tavares DA, Almeida ST, et al. The impact of private use of PCV7 in 2009 and 2010 on serotypes and antimicrobial resistance of Streptococcus pneumoniae carried by young children in Portugal: Comparison with data obtained since 1996 generating a 15-year study prior to PCV13 introduction. Vaccine. 2016;34(14):1648-56.

85. Ouldali N, Cohen R, Levy C, Gelbert-Baudino N, Seror E, Corrard F, et al. Pneumococcal susceptibility to antibiotics in carriage: a 17 year time series analysis of the adaptive evolution of non-vaccine emerging serotypes to a new selective pressure environment. Journal of Antimicrobial Chemotherapy. 2019;74(10):3077-86.

86. Palmu AA, Rinta-Kokko H, Nohynek H, Nuorti JP, Jokinen J. Impact of National Ten-Valent Pneumococcal Conjugate Vaccine Program on Reducing Antimicrobial Use and Tympanostomy Tube Placements in Finland. Pediatr Infect Dis J. 2018;37(1):97-102.

87. Ribitzky-Eisner H, Minuhin Y, Greenberg D, Greenberg N, Chodick G, Craiu M, et al. Epidemiologic and Microbiologic Characteristics of Occult Bacteremia Among Febrile Children in Southern Israel, Before and After Initiation of the Routine Antipneumococcal Immunization (2005-2012). Pediatr Neonatol. 2016;57(5):378-84.

88. Roddy MG, Glazier SS, Agrawal D. Pediatric mastoiditis in the pneumococcal conjugate vaccine era: symptom duration guides empiric antimicrobial therapy. Pediatr Emerg Care. 2007;23(11):779-84.

89. Shapiro DJ, Gonzales R, Cabana MD, Hersh AL. National trends in visit rates and antibiotic prescribing for children with acute sinusitis. Pediatrics. 2011;127(1):28-34.

90. Stevens RW, Wenger J, Bulkow L, Bruce MG. Streptococcus pneumoniae non-susceptibility and outpatient antimicrobial prescribing rates at the Alaska Native Medical Center. Int J Circumpolar Health. 2013;72:22297.

91. Stolbrink M, Bonnett LJ, Blakey JD. Antibiotics for COPD exacerbations: does drug or duration matter? A primary care database analysis. BMJ Open Respiratory Research. 2019;6(1):e000458.

92. Tamir SO, Roth Y, Dalal I, Goldfarb A, Grotto I, Marom T. Changing trends of acute otitis media bacteriology in central Israel in the pneumococcal conjugate vaccines era. Pediatr Infect Dis J. 2015;34(2):195-9.

93. Tief F, Hoppe C, Seeber L, Obermeier P, Chen X, Karsch K, et al. An inception cohort study assessing the role of pneumococcal and other bacterial pathogens in children with influenza and ILI and a clinical decision model for stringent antibiotic use. Antivir Ther. 2016;21(5):413-24.

94. Vasoo S, Singh K, Hsu LY, Chiew YF, Chow C, Lin RT, et al. Increasing antibiotic resistance in Streptococcus pneumoniae colonizing children attending day-care centres in Singapore. Respirology. 2011;16(8):1241-8.

95. Waddle E, Jhaveri R. Outcomes of febrile children without localising signs after pneumococcal conjugate vaccine. Arch Dis Child. 2009;94(2):144-7.

96. Zhou F, Shefer A, Kong Y, Nuorti JP. Trends in acute otitis media-related health care utilization by privately insured young children in the United States, 1997-2004. Pediatrics. 2008;121(2):253-60.

97. Dohna-Schwake C, Podlewski P, Voit T, Mellies U. Non-invasive ventilation reduces respiratory tract infections in children with neuromuscular disorders. Pediatr Pulmonol. 2008;43(1):67-71.

98. Mahamat A, Daurès JP, de Wzieres B. Additive preventive effect of influenza and pneumococcal vaccines in the elderly: results of a large cohort study. Hum Vaccin Immunother. 2013;9(1):128-35.
